# Ultra-conformal drawn-on-skin electronics for multifunctional motion artifact-free sensing and point-of-care treatment

**DOI:** 10.1038/s41467-020-17619-1

**Published:** 2020-07-30

**Authors:** Faheem Ershad, Anish Thukral, Jiping Yue, Phillip Comeaux, Yuntao Lu, Hyunseok Shim, Kyoseung Sim, Nam-In Kim, Zhoulyu Rao, Ross Guevara, Luis Contreras, Fengjiao Pan, Yongcao Zhang, Ying-Shi Guan, Pinyi Yang, Xu Wang, Peng Wang, Xiaoyang Wu, Cunjiang Yu

**Affiliations:** 1 Department of Biomedical Engineering, University of Houston, Houston, TX 77204 USA; 2 Department of Mechanical Engineering, University of Houston, Houston, TX 77204 USA; 3 Ben May Department for Cancer Research, The University of Chicago, Chicago, IL 60637 USA; 4 Materials Science and Engineering Program, University of Houston, Houston, TX 77204 USA; 5 Department of Chemistry, Ulsan National Institute of Science and Technology (UNIST), Ulsan, 44919 Republic of Korea; 6 Department of Electrical and Computer Engineering, University of Houston, Houston, TX 77204 USA; 7 Texas Center for Superconductivity, University of Houston, Houston, TX 77204 USA

**Keywords:** Electrical and electronic engineering, Sensors and biosensors

## Abstract

An accurate extraction of physiological and physical signals from human skin is crucial for health monitoring, disease prevention, and treatment. Recent advances in wearable bioelectronics directly embedded to the epidermal surface are a promising solution for future epidermal sensing. However, the existing wearable bioelectronics are susceptible to motion artifacts as they lack proper adhesion and conformal interfacing with the skin during motion. Here, we present ultra-conformal, customizable, and deformable drawn-on-skin electronics, which is robust to motion due to strong adhesion and ultra-conformality of the electronic inks drawn directly on skin. Electronic inks, including conductors, semiconductors, and dielectrics, are drawn on-demand in a freeform manner to develop devices, such as transistors, strain sensors, temperature sensors, heaters, skin hydration sensors, and electrophysiological sensors. Electrophysiological signal monitoring during motion shows drawn-on-skin electronics’ immunity to motion artifacts. Additionally, electrical stimulation based on drawn-on-skin electronics demonstrates accelerated healing of skin wounds.

## Abstract (web summary)

Designing efficient wearable bioelectronics for health monitoring, disease prevention, and treatment, remains a challenge. Here, the authors demonstrate an ultra-conformal, customizable and deformable drawn-on-skin electronics which is robust to motion artifacts and resistant to physical damage.

## Introduction

Human skin offers a wealth of physiological and physical information that can be utilized to monitor, prevent, and treat adverse health conditions. Through directly interfacing electronic devices with the skin, information such as the state of the heart, the condition of muscle, the impedance, and hydration of the skin, can all be extracted^1^. The past decade has witnessed fast advances in wearable bioelectronics as a viable technology for epidermal sensing. Typically, wearable bioelectronics are structured in the form of patches that are soft, flexible, and/or stretchable^2–4^ and thus, are beneficial to form contact with the curvilinear surfaces of the skin. However, wearable bioelectronics generally suffer from motion artifacts leading to misinterpretation and misdiagnoses, which is mainly due to the weak adhesion or imperfect conformability, and thus inconsistent interface between the electronics and skins^5–10^. Specifically, one of the main sources of motion artifacts lies in mechanical disturbances at the electronics-skin interfaces^11,12^.

Here, we present ultra-conformal Drawn-on-Skin (DoS) electronics as a new bioelectronics platform for on-demand multifunctional, motion artifact-free sensing. Compared to existing wearable and/or printed bioelectronics fabricated based on dedicated equipment^13–17^, DoS electronics has numerous advantages including: simple fabrication without dedicated equipment, ability to deposit electronic materials to dynamic surfaces, capability to construct active electronics, multifunctionality of devices and sensors, immunity to motion artifacts without the need for additional hardware or computation, which offers an unprecedented solution to the long-standing challenge in the bioelectronics field, and customizability for personalized point-of-care treatment. Specifically, DoS electronics is created by liquid functional inks drawn into stencils using ballpoint pens directly on human skin. Upon drawing, an ultra-conformal, robust, and stretchable interface that is immune to motion is formed between DoS electronics and skin. The DoS electronic devices are based on the Ag flakes/poly(3,4-ethylenedioxythiophene)-poly(styrenesulfonate) (Ag-PEDOT:PSS) composite, poly(3-hexylthiophene-2,5-diyl) nanofibrils (P3HT-NF), and ion gel as the conductive, semiconducting, and dielectric inks, respectively. As a versatile platform, DoS electronics devices such as thin-film transistors, strain sensors, temperature sensors, heaters, hydration sensors, and electrophysiological (EP) sensors have been developed with features of skin-textured surface, curvilinear shape, and mechanical deformability. A wireless DoS electrocardiogram (ECG) monitoring system demonstrates daily and clinical usages. By comparing the DoS EP sensors with hospital-grade gel electrodes, and ultrathin serpentine mesh electrodes, we found that DoS electronics has multiple advantages, such as stable performance in the presence of sweat, reliable capture of EP signals over a long duration, strong adherence to the skin, and immunity to motion artifacts during sensing. Accelerated skin wound healing from DoS electronics-based electrical stimulation also illustrates its usage for point-of-care treatment. Studies of the materials and device design, fabrication, characterization, and applications in motion artifact-free sensing and wound healing portray the crucial capabilities and applicability of the DoS electronics platform.

## Results

### DoS electronic inks

**Fig. 1:**
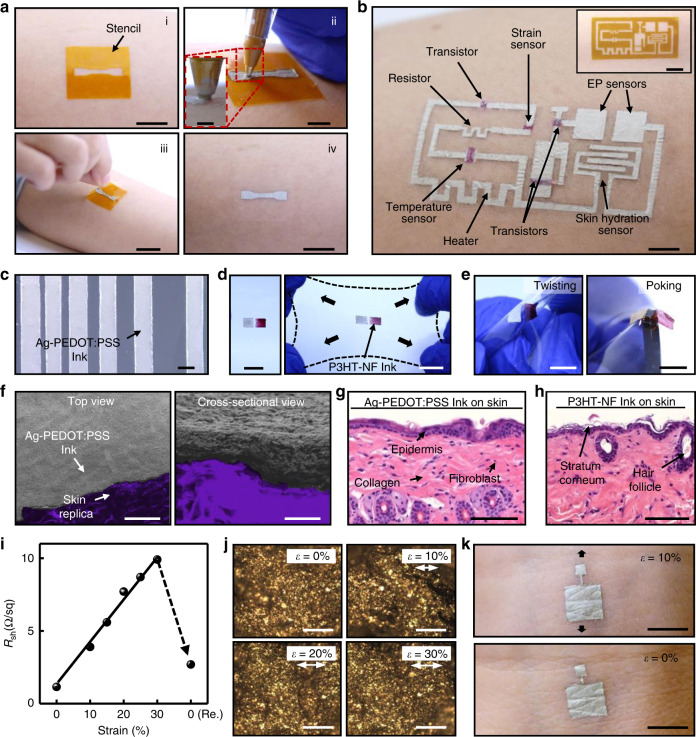
DoS electronics platform featuring conductive and semiconducting inks. **a** DoS electronics drawing process beginning with (i) a tape-based stencil attachment to the skin (scale bar 1 cm), (ii) drawing the device with the stencil, modified ballpoint pen, and inks (scale bar 5 mm), inset is a closeup of the tip (scale bar 1 mm), (iii) removal of the stencil (scale bar 2 cm), and (iv) the completed device after drying (scale bar 1 cm). **b** An example DoS integrated system including a resistor, transistors, heater, EP (electrophysiological) sensors, temperature sensor, strain sensor, and skin hydration sensor drawn on human skin (scale bar 5 mm). The corresponding stencil for the conductor is shown in the inset (scale bar 1 cm). **c** Achievable line spacing using stencils and the conductive Ag-PEDOT:PSS ink (scale bar 1 mm). **d** Non-stretched (left, scale bar 1 mm) and biaxially stretched (right, scale bar 1 cm) states of the drawn structure consisting of Ag-PEDOT:PSS conductive (left square) and P3HT-NF semiconducting (right square) inks on PDMS. **e** Twisting (left) and poking (right, scale bars 1 cm) deformations of the drawn structure with conductive and semiconducting inks. **f** SEM images of the Ag-PEDOT:PSS ink showing ultraconformal contact with the grooves and bumps (left, scale bar 250 µm) on top of skin replica and cross-section showing Ag-PEDOT:PSS microstructure (right, scale bar 20 µm). The purple color indicates the skin replica. **g** Histology image of the Ag-PEDOT:PSS ink on skin of mice after 48 h (scale bar 100 µm). **h** Histology image of the P3HT-NF ink on skin of mice after 48 h (scale bar 100 µm). **i** Sheet resistance vs. strain while stretching the Ag-PEDOT:PSS ink up to 30% and releasing. The error bars represent the s.d. **j** Optical microscope images of the Ag-PEDOT:PSS ink on skin replica stretched up to 30% (scale bars 100 µm). **k** DoS EP sensor on the wrist of subject in stretched (top) and non-stretched (bottom) states (scale bars 5 mm).

The conductive ink was prepared by mixing Ag flakes with PEDOT:PSS solution (Supplementary Fig. 1a, b). The semiconducting ink shown in Supplementary Fig. 1c, d and ion gel ink were also prepared as mixtures. Details for all the ink preparations can be found in the Methods section. The DoS electronics drawing process is illustrated in Fig. 1a. First, a stencil based on Kapton and clear tape (Magic Tape, 3 M) was made with a cutting machine (Silhouette Cameo) and adhered to the target skin. The inks were then drawn into the outlines of the stencils using a modified ballpoint pen (see Supplementary Information) with a tip diameter of 1 mm (Supplementary Fig. 1e). It is noted that the tip of the pen does not need to touch the skin for the ink to be drawn since a meniscus is formed, as shown in the inset of Fig. 1a. The motion of the pen induces shear on the meniscus, causing the ink to spread across the skin surface. The speed of drawing was ~10 mm/s. After ~ 5 min of solvent evaporation at room temperature, the stencil was removed, and a dry thin film remained. If there are any imperfections in the DoS device, they can be corrected by drawing over the imperfections (Supplementary Fig. 1f).

The morphologies of the resulted films created by drawing with the inks were investigated. The scanning electron microscope (SEM) image (Supplementary Fig. 2) of the conductive ink shows nano/microscale Ag flakes and the atomic force microscope (AFM) images (Supplementary Fig. 3a, b) of the semiconducting ink show nanofibrous structures of P3HT. To demonstrate the versatility of the DoS electronics platform, a prototype multifunctional integrated system (Fig. 1b) was drawn to show that several devices, including transistors, strain sensor, temperature sensor, heater, resistor, skin hydration sensor, and EP sensors, can be made using the drawing process. The inset shows the corresponding stencil for the Ag-PEDOT:PSS ink and the fabrication of the integrated system is described further in the Supplementary Information. The DoS integrated system could undergo stretching and compressing without any physical damages, as portrayed in Supplementary Fig. 4.

The ink drawing characteristics, mechanical properties, skin compatibility, and electrical performances of the Ag-PEDOT:PSS and P3HT-NF inks were evaluated. The ink line width and resolution could be controlled by varying the tip diameter of the pen or by using stencils. The Ag-PEDOT:PSS ink line width was ~ 1 mm but could be reduced to 300 µm (Supplementary Fig. 5) by using a pen with a finer tip. A stencil could reduce the line spacing to 200 µm without creating intersecting lines (Fig. 1c). P3HT-NF and ion gel inks, shown in Supplementary Fig. 6a, b, respectively, were drawn into lines to demonstrate the writability of the inks. Multiple variants of the Ag-PEDOT:PSS ink were prepared and compared to determine the optimal ratio of 1: 2 (Ag flakes: PEDOT:PSS solution), as shown in Supplementary Fig. 7: this is further explained in the Methods section. The drying time ranged from 3–5 min (Supplementary Fig. 8a). Although the DoS electronics drawing process involves manual control, different thicknesses (few hundred nm to 10 µm) of the Ag-PEDOT:PSS ink layer could be achieved by drawing multiple times over the same location (Supplementary Fig. 8b). It is worth mentioning that the pen can be engineered to allow better control of the ink flow and the ink/skin interface to ensure the ink uniformity and repeatability, thus eliminating the potential device variation issue.

To examine the robustness of both the Ag-PEDOT:PSS ink and P3HT-NF inks to various modes of deformation, they were drawn onto polydimethylsiloxane (PDMS), shown in Fig. 1d (left). Biaxial stretching (~20%) shows almost no cracks in the drawn structure (Fig. 1d, right). Neither twisting nor poking caused visible damage, as shown in Fig. 1e. A colorized SEM image of the Ag-PEDOT:PSS ink on a PDMS based skin replica (colored in purple) shows that the ink fills in and conformally lays on the grooves and valleys (Fig. 1f, left). The cross-sectional view provides confirmation and shows the microstructure of the Ag-PEDOT:PSS ink (Fig. 1f, right). The fabrication of the PDMS skin replica is described in the Supplementary Information.

In order to verify the skin response to the Ag-PEDOT:PSS and P3HT-NF inks, they were drawn onto the backs of mice after removing the fur, then the skin samples were extracted and evaluated through histological staining procedures. Compared to the control (non-ink treated) skin sample (Supplementary Fig. 9), the 48-h skin samples with drawn Ag-PEDOT:PSS and P3HT-NF inks (Fig. 1g, h, respectively) showed no malignancies or inflammation in the epidermis, or in the dermis underneath. The Ag-PEDOT:PSS ink shows low resistivity with a sheet resistance of 1.2 Ω/sq under no strain and 9.9 Ω/sq under 30% strain (Fig. 1i). Releasing the strain resulted in a decrease to 2.7 Ω/sq. After 1000 cycles of stretching at 10% strain, the sheet resistance of the Ag-PEDOT:PSS ink slightly increased (Supplementary Fig. 10). Storing the Ag-PEDOT:PSS ink in the ambient environment (22 °C) or in the fridge (4 °C) did not affect its resistance over a couple of months, as shown in Supplementary Fig. 11. Optical microscope images of the Ag-PEDOT:PSS ink on the PDMS skin replica substrate show that no obvious crack was observed up to 30% strain (Fig. 1j). It is noted that the PDMS surface was made more hydrophilic (Supplementary Fig. 12a, b) for improved adhesion, but those treatments are not required for actual skins as they are partially hydrophilic^9^. Figure 1k shows the skin-textured morphology of the DoS EP sensor with (top frame) and without (bottom frame) stretch on human skin, indicating the ultra-conformable and deformable nature of the DoS devices.

### DoS electronic devices and sensors on skin replica

**Fig. 2:**
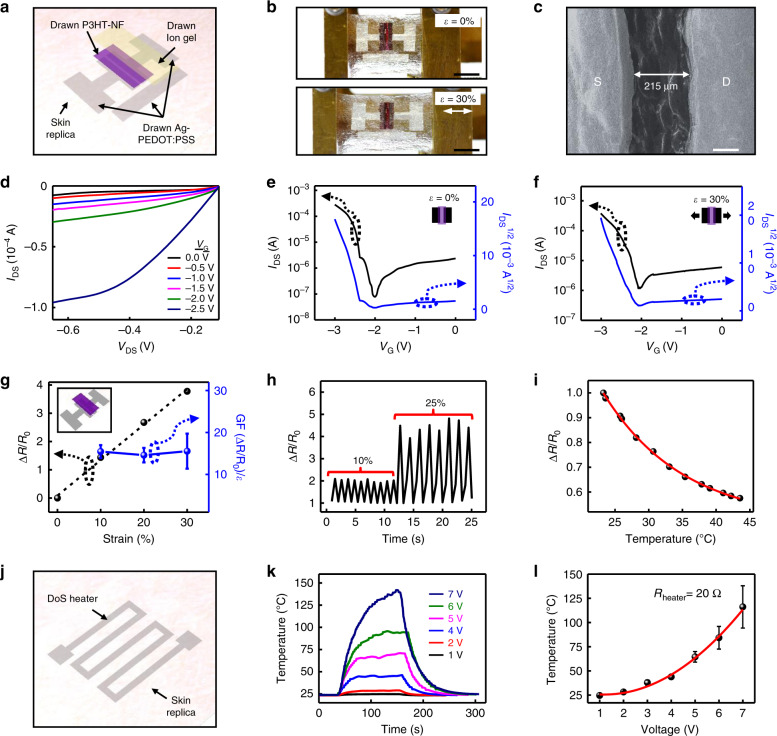
DoS electronic devices and sensors on skin replica. **a** Schematic of the DoS transistor based on the Ag-PEDOT:PSS ink as the conductor, P3HT-NF ink as the semiconductor, and ionic gel ink as the dielectric. **b** DoS transistor on skin replica under no strain (top) and 30% strain (bottom, scale bars 5 mm). **c** SEM image of the drawn source (S) and drain (D) electrodes and the channel (scale bar 100 µm). **d** I-V curve of DoS transistor without applied strain. **e**, **f** Transfer curves of DoS transistor without and 30% strain along the transistor channel length direction. **g** Electrical resistance of the DoS strain sensor under applied mechanical strain up to 30% and the corresponding gauge factors. Inset shows a schematic of the DoS strain sensor. The error bars represent the s.d. **h** Relative resistance change of the DoS strain sensor under cyclic stretching and releasing at 10% and 25% strain. **i** Relative resistance change of the DoS temperature sensor under different temperature conditions. **j** Schematic of the DoS heater based on the conductive ink. **k** Temperature profiles under different applied voltage on the DoS heater. **l** Calibration curve of the DoS heater. The error bars represent the s.d.

To validate the performance of DoS electronics on uneven and rough surfaces, transistors, strain sensors, temperature sensors, and heaters were characterized on skin replicas. Transistors, which are fundamental building blocks in electronic circuits and biomedical devices and systems, were fabricated using the DoS electronics drawing process. The exploded schematic of the DoS transistor, composed of Ag-PEDOT:PSS ink as the conductor, P3HT-NF ink as the semiconductor, and ion gel ink as the dielectric, is shown in Fig. 2a. The fabrication of the DoS transistor using the corresponding stencils (Supplementary Fig. 13) is detailed in the Methods section. Images of the skin-textured transistor under no strain (top frame) and 30% strain (bottom frame) are presented in Fig. 2b. The channel length could be made on the order of a few hundred microns as shown in the SEM image in Fig. 2c. The I-V curves show typical p-type transistor characteristics (Fig. 2d). To obtain the transfer curves, the bias voltage, V_DS_, was set to −0.55 V and the gate voltage, V_G_, was swept from 0 to −3 V. The on/off ratio (I_ON_/I_OFF_) and mobility (µ_FE_) were calculated to be 1.69 × 10^3^ and 7.07 cm^2^ V^−1^ s^−1^ when the DoS transistor was under no strain, and 2.01 × 10^2^ and 5.36 cm^2^ V^−1^ s^−1^ under 30% strain, respectively (Fig. 2e, f). Some hysteresis could be observed, as shown in Supplementary Fig. 14, but this can be expected for electrolytically gated transistors^18^. The threshold voltage (V_TH_) underwent a slight decrease from −2.38 V under no strain to −2.03 V under 30% strain. The calculations for µ_FE_ are detailed in Supplementary Note 1. Though the drawing process is fully manual, these results indicate that it can be used to develop functional and stretchable DoS transistors, as a proof-of-concept. The DoS transistor could potentially be used in readout applications, interface electronics, signal amplification, or integrated circuits.

Strain sensors and temperature sensors, which can reveal critical physiological information from the skin^19,20^, were drawn in the format of two-terminal sensors as shown in the inset of Fig. 2g. Both devices were constructed using the Ag-PEDOT:PSS ink as the conductor and P3HT-NF ink as the semiconductor with the stencils shown in Supplementary Fig. 15. For the DoS strain sensor, static testing showed that the resistance change increased by a factor of 4 (Fig. 2g) after stretching up to 30%, while the gauge factor remained around 15 during the stretching process. The cyclical stretch and release test in Fig. 2h shows that the resistance change increased by a factor of 2 under 10% strain and a factor of 4.5 under 25% strain. The detailed gauge factor calculation is presented in Supplementary Note 2. The DoS temperature sensor showed that the resistance change decreased from 1 to 0.57 over the temperature range of 20–45 °C (Fig. 2i). The response of the DoS temperature sensor is similar to that of a negative temperature coefficient (NTC) thermistor. Based on the linear plot of ln(R) vs. 1000/T shown in Supplementary Fig. 16, the thermistor constant (*β)* was determined to be 2589 K, which is comparable to commercial NTC thermistors^21^. The calculation for *β* is shown in Supplementary Note 3. Ag-PEDOT:PSS ink was drawn into the stencil (Supplementary Fig. 17) to fabricate the DoS heater (schematically shown in Fig. 2j), which could potentially be used for skin wound healing^22,23^. The dynamic temperature change of the DoS heater was obtained by applying DC bias from 1 to 7 V to produce temperatures ranging from 25 to 140 °C, as plotted in Fig. 2k. The calibration curve for the DoS heater is shown in Fig. 2l.

### DoS electronic devices and sensors on skin

**Fig. 3:**
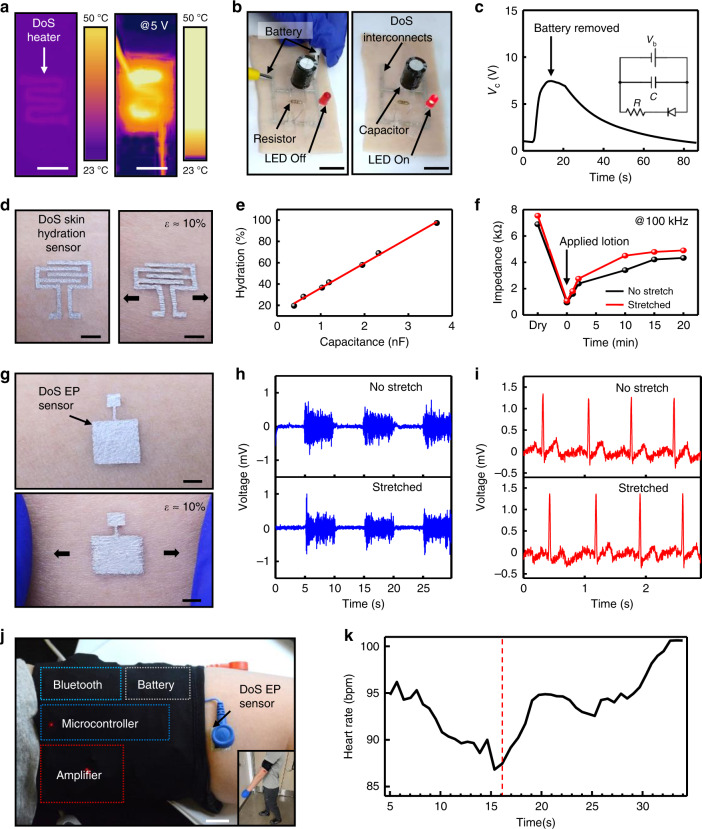
DoS electronics on skin and wireless sensing. **a** IR camera images of the DoS heater on skin without applied voltage (left) and with an applied voltage of 5 V (right, scale bars 1 cm). **b** Ag-PEDOT:PSS ink serving as the interconnection during charging of a capacitor (left) and discharging through the resistor and LED (right, scale bars 2 cm). **c** Voltage of the capacitor showing charging until the battery is removed and then discharging afterwards. Inset is the circuit diagram showing a battery connected to a capacitor, which is connected to a resistor and LED that are connected in series. **d** DoS skin hydration sensor on skin without (left) and with applied strain (right, scale bars 2 mm). **e** Calibration of the DoS skin hydration sensor using a commercial hydration meter. **f** Impedance of the DoS skin hydration sensor without and with mechanical stretch. The arrow indicates the time at which lotion was applied to the dry skin. **g** DoS EP sensors without (top) and with applied strain (bottom, scale bars 2 mm). **h** Recorded EMG signals without (top) and with applied strain (bottom). **i** Recorded ECG signals without (top) and with mechanical stretch (bottom). **j** Schematic of the wireless transmission circuit components mounted on the arm and interface with the DoS EP sensors (scale bar 2 cm). **k** Average beats/min over all trials derived from the ECG signals attained with the DoS EP sensors. In the time prior to the red dashed line, the subject was standing still. After the red dashed line, the subject began walking.

The Ag-PEDOT:PSS ink was utilized to construct a heater, interconnection for an RC circuit, skin hydration sensor, and EP sensors on porcine and human skin. Images from an infrared camera (FLIRONE Pro, FLIR® Systems, Inc.) of the DoS heater show the high conductivity of the ink on the rough and uneven surface of porcine skin (Fig. 3a). To demonstrate another use of the ink on skin, electronic components were arranged into an RC circuit with an LED and wired with Ag-PEDOT:PSS ink as the interconnection (Fig. 3b, left). A capacitor (C = 2200 µF) was charged with a battery (V_b_ = 9 V) connected to the circuit diagrammed in the inset of Fig. 3c. After removing the battery, the capacitor discharged current through the rest of the circuit, which flowed through a resistor (R = 800 Ω) and turned on an LED (Fig. 3b, right). Common medical sensors were also fabricated with the Ag-PEDOT:PSS ink.

The DoS skin hydration sensor was constructed in the form of two interdigitated electrodes with the appropriate stencil (Supplementary Fig. 18) to record skin impedance, from which the hydration level of the epidermis and dermis can be extrapolated. The interdigitation allows for greater interaction between the two electrodes and increased capacitive sensing capability^24^. The skin-textured surface of the DoS skin hydration sensor can be clearly observed in its non-stretched (Fig. 3d, left) and stretched (Fig. 3d, right) states. The linear relationship shown in Fig. 3e (*R*^2^ = 0.997) between the commercial hydration meter (units of % hydration) measurements and those of the drawn skin hydration sensor (capacitance, units of nF) can be written as

$$\% H = bC + a$$

where *%H* is the percent skin hydration, *C* is the measured capacitance, *b* is the slope, and *a* is the calibration constant. After lotion was applied to the skin, the impedance sharply decreased^25^, indicating that the DoS skin hydration sensor could detect the change in hydration of the skin, even when stretched (Fig. 3f). Additional impedance data, details of the interface, and measurement procedure are shown Supplementary Fig. 19a–f and are described further in Supplementary Note 4. The Ag-PEDOT:PSS ink also served as a viable EP sensor, depicted in Fig. 3g in non-stretched (top) and stretched (bottom) states. The DoS EP sensors, when drawn in the appropriate locations using stencils (Supplementary Fig. 20), could capture electromyographic (EMG) and ECG signals, as demonstrated in Fig. 3h, i, respectively. For EMG acquisition, two EP sensors were drawn on the forearm and bicep of the same arm (Supplementary Fig. 21a). To capture ECG signals, one EP sensor was drawn on each wrist of the subject (Supplementary Fig. 21b), and then connected to a data acquisition system (DAQ). Further details are in the Methods section. The signal-to-noise ratio (SNR) of the ECG signals recorded with the DoS electrodes remains approximately the same (~45 dB) even after stretching. The details of the SNR calculation for the ECG signals are in Supplementary Note 5 and the power spectral density of the signal is shown in Supplementary Fig. 22. Additionally, the DoS sensors and devices could be encapsulated (Supplementary Fig. 23a, b) for protection against wet environments and still retain their functions, such as ECG recording (Supplementary Fig. 23c). It is important to note that all the EP signals shown in this work have not been postprocessed. As shown with the previous sensors, the EP sensors function on the skin even when under strain, with no observable changes in the acquired signals.

### Wireless ECG monitoring with DoS electronics

Fully portable EP signal monitoring enables real-world and clinical usage of DoS electronics. The DoS EP sensors were interfaced with a custom-built wireless transmission circuit, schematically shown in Fig. 3j. Details of the setup and interface are in the Methods section and are shown in Supplementary Fig. 24a–c. As walking is considered to be one of the most representative daily activities and is a part of clinical stress tests^26–28^, a subject was instructed to stand still for 16 s and rest, and then walk for 16 s (inset of Fig. 3j) for a simple stress test. An obvious decrease in HR can be observed (in Fig. 3k) as the subject stood still and relaxed (average HR was 91.5 beats per minute, bpm), but then it rose (average HR was 95.1 bpm) as the subject walked. Additional details for the experiment and processing are in the Methods section. The detection of the change in HR indicates that the DoS EP sensors are compliant for cardiac diagnostics. Furthermore, a representative trial of ECG data recorded during the test is shown in Supplementary Fig. 25a, b, shows distinguishable P, QRS, and T sub-waves even during walking, signifying the capability for wireless DoS EP monitoring system to sufficiently capture ECG signals during regular activity and clinical examinations.

### Comparisons of DoS electronics with existing technologies

**Fig. 4:**
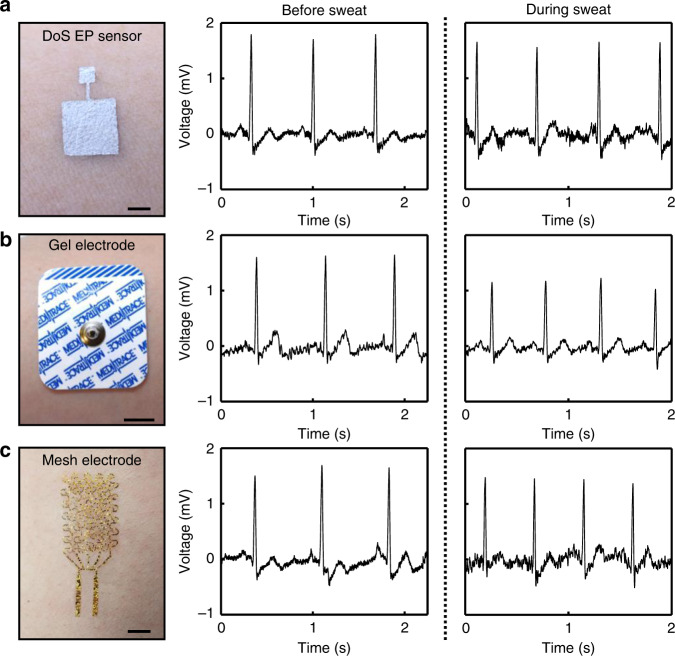
Comparison of DoS electronics and existing technologies for ECG sensing before and during sweat conditions. **a** DoS EP sensor (left, scale bar 2 mm) with ECG signals recorded before sweating (middle) and while sweating (right). **b** Gel electrode (left, scale bar 1 cm) with ECG signals recorded before sweating (middle) and while sweating (right). **c** Mesh electrode (left, scale bar 5 mm) with ECG signals recorded before sweating (middle) and while sweating (right).

To determine the advantages of the DoS EP sensors amongst the existing sensor technologies, they were compared to both hospital-grade gel electrodes (Meditrace 450 Foam Electrodes, Kendall) and ultrathin (350 nm thick) serpentine mesh electrodes for their performance in the presence of sweat, durability over time, adhesion to the skin, and robustness against motion artifacts. The details of the sweat, durability over time, and adhesion tests are described in the Methods section. DoS electrodes were fabricated as mentioned before. The hospital-grade gel electrodes were placed on the skin without modification. The mesh electrodes were designed and fabricated using microfabrication techniques (Supplementary Information, schematically illustrated in Supplementary Fig. 26) and the detailed geometrical design and schematic of the interface with the skin are shown in Supplementary Fig. 27 and Supplementary Fig. 28, respectively. Two sensors of each type were drawn/placed on the wrists of the subject for ECG recording. The ECG signals attained with the DoS EP sensors are comparable to those of the other sensor types prior to sweating (Fig. 4a–c, middle). The ECG signals recorded while sweating using the DoS EP sensors and mesh electrodes remained relatively consistent with those recorded prior to sweating, unlike the signals recorded using the gel electrodes (Fig. 4a–c, right). Specifically, a dampened ECG signal was observed for the gel electrodes, which is a result of sweat pooling or a decrease in ion concentration in the gel electrolyte^29,30^. Sweating minimally affected the physical appearance of the DoS EP sensor, as shown in Supplementary Fig. 29. The SNRs of the sensors before and during sweating (Supplementary Table 1) show that the DoS EP sensors and mesh electrodes show little change (~1 dB). However, the SNR for the gel electrodes after sweating decreased by ~3 dB, which coincides with the noticeable amplitude decrease. Furthermore, all three types of sensors were evaluated for their durability over the course of 7 h. The ECG signals attained with the DoS EP sensors were relatively consistent over all the measurement time points, unlike the other sensors (Supplementary Fig. 30a–c).

The skin adhesion of the sensors was also examined through peeling and rubbing tests. As shown in Supplementary Fig. 31a–e, the DoS EP sensor cannot be removed from the skin, whereas the others were easily removed. The DoS EP sensors remained intact when rubbed vigorously while the mesh electrodes were irreversibly damaged (Supplementary Fig. 32a, b, respectively). Any imperfections in the DoS devices could quickly be fixed as mentioned before, whereas an entirely new device would be necessary for the damaged mesh electrodes.

### Motion artifact-free EP sensing on human skin

Motion artifacts are particularly problematic in wearable health monitoring and lead to misinterpretation and misdiagnoses that have severe health implications^31,32^. Studies have shown that misdiagnosis due to motion artifacts can substantially reduce quality of life for patients^33,34^. Artifacts that originate from electrode-skin disturbances occur because of the electrode’s improper adhesion to skin during movement^6,7,35^. To evaluate the response of the three types of sensors to induced motions, two experiments were conducted including local skin deformation while recording the ECG signals and vibration of the arm while recording the resting EMG signals. It is noted that any obvious deviations from the sinus ECG waveform during deformation or resting EMG potentials during vibration in each of the respective experiments were considered as motion artifacts.

In order to validate the response of the three sensor types to skin deformation, the ECG from a human subject was recorded with regular intervals of stretching, compressing, and releasing the skin at the site of one of the sensors, for each sensor type. The same wired data DAQ setup (Supplementary Fig. 33a) was used as mentioned before and an example deformation is shown in Supplementary Fig. 33b. Further details are in the Methods section. It should be noted that this method of deforming the skin under the electrode served as an extreme version of the deformations the skin experiences under day-to-day motions.

**Fig. 5:**
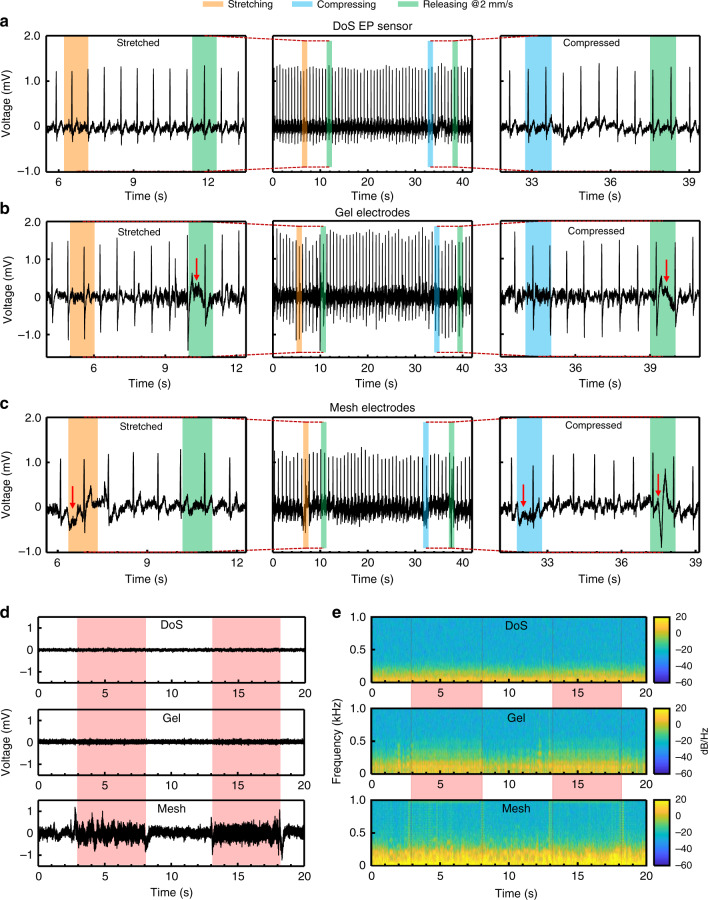
Motion artifact-free sensing with DoS electronics. **a** Recorded ECG signals (middle) from the DoS EP sensors during local stretching/releasing cycle (left) and compressing/releasing cycle (right). The orange bars indicate the duration of the stretching motion, the blue bars indicate the duration of the compressing motion, and the green bars indicate the duration of the releasing motion. **b** Recorded ECG signals (middle) from the gel electrodes during local stretching/releasing cycle (left) and compressing/releasing cycle (right). The red arrows indicate artifacts. **c** Recorded ECG signals (middle) from the mesh electrodes during local stretching/releasing cycle (left) and compressing/releasing cycle (right). The red arrows indicate artifacts. **d** Resting EMG signals recorded with vibration-induced motion of the arm. The pink bars indicate the duration of time in which the VM was turned on for the DoS EP sensors (top), gel electrodes (middle), and mesh (bottom) electrodes. **e** TF maps of the resting EMG signals with the vibration-induced motion for the DoS EP sensors (top), gel electrodes (middle), and mesh (bottom) electrodes. The pink bars and red lines indicate the duration of the vibrations.

Representative trials of deforming the skin while recording ECG with the DoS EP sensors, gel electrodes, and mesh electrodes are shown in Fig. 5a–c, respectively. The regions of interest are highlighted in the corresponding colors of orange, blue, and green, with each color representing stretching, compressing, and releasing motions, respectively (Fig. 5a). As shown in Fig. 5a, the signal recorded from the DoS EP sensors during the motions shows no abnormal deviations from the sinus ECG waveform. The gel electrodes show that the deformations occasionally distorted the signal (red arrows in Fig. 5b). These artifacts likely originated due to sliding at the gel electrolyte-skin interface induced by the manually applied skin deformation^36,37^. The mesh electrodes also show signal fluctuations during the deformations (red arrows in Fig. 5c). Sliding between the mesh electrode and skin during the deformations occurred due to the inconsistent adhesion of the mesh electrode to the skin^5^. Zoomed in views of these data from the three sensor types are in Supplementary Figs. 34–36, respectively. SNR values were calculated for the signals shown in the middle frames of Fig. 5a–c and were determined to be 50 dB, 20 dB, and 12 dB for the DoS EP sensors, gel, and mesh electrodes, respectively. Supplementary Fig. 37a, b shows how the noise and artifacts were isolated for the gel and mesh electrodes, respectively, and further description is in Supplementary Note 6. Clearly, the noise and motion artifacts can substantially affect the SNR. The results for the DoS EP sensors were further verified with a custom-made stretching apparatus for reproducible skin deformations (Supplementary Fig. 38a, b). The details are presented in the Supplementary Information. As expected, no artifacts could be observed in the ECG signals during the deformations produced by the custom-made stretching apparatus (Supplementary Fig. 39a, b).

In addition to characterizing the ECG signals during the skin deformations, the change in skin to electrode impedance (SEI) during the deformations was evaluated, as it has been associated with motion artifacts^38^. Supplementary Fig. 40a illustrates the circuit diagram of the setup that was adopted to acquire the SEI changes^39,40^. Supplementary Note 7 provides details regarding the setup and experimental parameters. In addition, the impedance (and resistance) normalized by contact area is shown in Supplementary Table 1. The magnitude of the change in SEI (shown in Supplementary Fig. 40b–d) during all deformations including stretching, compressing, and releasing were determined to be 138 ± 41.5 Ω, 54.1 ± 13.2 Ω, and 1.81 ± 1.44 kΩ, for the DoS EP sensors, gel electrodes, and mesh electrodes, respectively. Statistical analysis (Supplementary Note 7) showed no significant difference (*P* = 0.99) between the average change in SEI for the DoS EP sensors and gel electrodes. These data indicate that the change in SEI for the DoS EP sensors and gel electrodes was not a substantial contributor to the results shown in Fig. 5a–c. Furthermore, impedance changes on the surface of the sensor during stretching and releasing on human skin were obtained (Supplementary Fig. 40e). Supplementary Note 7 contains details of the experimental setup. The change in the impedance during the deformations was minimal over various measurement frequencies relevant to EP signal monitoring^41,42^, which makes the DoS EP sensors well-suited for recording biological signals in the presence of skin deformations.

Other studies that investigated the origins of motion artifacts induced motions in the z-direction, or in the direction perpendicular to the surface of the skin, by attaching a vibrating motor (VM) near the sensor to simulate movement of the arm^7,43–45^. Since it has been shown that thinner sensors are less susceptible to motion artifacts^7^ and the DoS EP sensors can be as thin as a few 100 nm, the response of DoS EP sensors to vibration-induced motion (setup shown schematically in Supplementary Fig. 41) was investigated. The responses of the gel and mesh electrodes were also evaluated and details are in the Methods section. From the time domain resting EMG signals (Fig. 5d), the DoS EP sensors (top) and gel electrodes (middle) show no obvious susceptibility to the vibration, whereas the mesh electrodes (bottom) are substantially affected. Although the mesh was ultrathin, a higher amplitude of the vibration could produce artifacts that could be captured at the electrode sites^7^. Zoomed-in views of the time domain signals at the VM on and off states in Supplementary Fig. 42a–d suggest that the DoS EP sensors and gel electrodes are both unaffected by this vibration. However, further analysis in the time-frequency domain revealed information that could not be observed from only the time domain signals.

The time-frequency (TF) maps in Fig. 5e were constructed to analyze the frequency components of the signals. The resting EMG signals (<250 Hz) indicated by the yellow (20 dB) color can be seen throughout the TF maps of all the sensors. The mesh electrodes (bottom) were directly impacted by the vibration, which is indicated by the green (representing −10 to −20 dB) color near 1 kHz. Although the VM vibrated at 1 kHz, there was a substantial presence of lower frequency (>250 and <500 Hz) signals throughout the entire examined spectrum during the vibration, shown in the TF map for the mesh electrodes. This lower frequency content is also prevalent in the TF map for the gel electrodes when the VM is on and is clearly distinguishable as compared to when the VM was off, indicating that the gel electrodes were actually susceptible to the vibration. Unlike both the gel and mesh electrodes, the DoS EP sensors do not show this artifact in the time-frequency domain as can be seen in the corresponding TF map (Fig. 5e, top). It is noted that spikes observed in the signals as the VM turned on and off were not considered as artifacts generated by vibration. Further experiments validated the results for the DoS EP sensors. Neither increasing the vibration amplitude of the VM nor moving the VM closer to the DoS EP sensors produced artifacts in the recorded EMG signals as shown in Supplementary Fig. 43a–e.

### Accelerated skin wound healing with DoS electronics

**Fig. 6:**
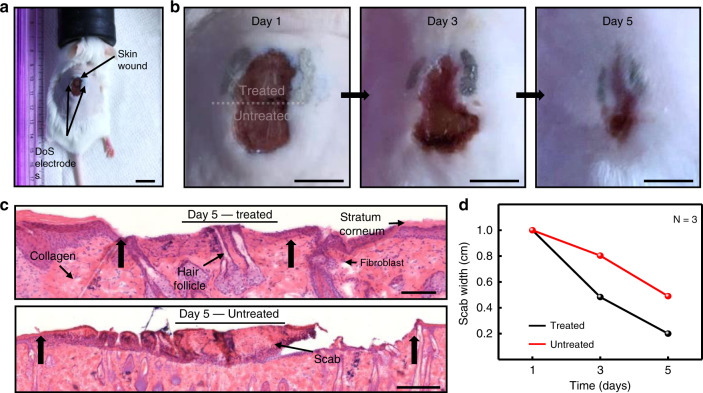
Accelerated skin wound healing with DoS electronics. **a** Experimental setup showing DoS electrodes around a skin wound on the back of mice (*N* = 3). The DoS electrodes served as conductive paths for electrical stimulation (scale bar 2 cm). **b** Photos of the wound healing on day 1 (left), day 3 (middle) and day 5 (right). Top half of the wound was treated with electrical stimulation while the bottom half was left untreated and healed naturally (scale bar 1 cm). **c** Histology images of the treated (top, scale bar 100 µm) and untreated (bottom, scale bar 250 µm) halves of the wound on day 5. The black arrows indicate the width of the wound. **d** Scab width over time for the treated (black) and untreated (red) halves of the wound.

As mentioned previously, the simplicity of the DoS electronics drawing process renders the technological platform suitable for low-resource areas. Another clear advantage is that the DoS electrodes could be customized to any wound shape and size. For instance, soldiers on the battlefield frequently experience skin wounds from shrapnel^46^, which can take a few days to months to fully heal. The application of pulsed electrical stimulation for skin wound healing has been shown to advance wound recovery, particularly in the phases of proliferation and remodeling^22,47–49^. A mice skin wound model was employed, and electrical stimulation through DoS electronics showed accelerated wound healing. A skin wound was surgically created on the back of mice (*N* = 3) and electrodes were drawn around the top half of the wound, as shown in Fig. 6a, b, respectively. The stimulation setup is schematically shown in Supplementary Fig. 44 and experimental details can be found in the Methods section. Histology images show that the treated half shows no visible scab (Fig. 6c, top), although visual observation by eye suggested that a thin layer of scab was present. The untreated half shows a large scab (Fig. 6c, bottom), in contrast to the treated side which indicated slower natural healing. The histology images also show that the cross-section of the treated half had a narrower wound width (indicated by the black arrows) that that of the non-treated half. Additionally, the wound (scab) width was measured on days 1, 3, and 5. The scab width decreased from 1 cm to 0.49 cm (untreated) and to 0.20 (treated) by day 5, clearly demonstrating accelerated healing for the treated half (Fig. 6d). Interfacing a miniature DC stimulator to the DoS electronics would be extremely advantageous for on-the-go treatment. The accelerated wound healing results highlight the performance and customizability of DoS electronics for medical treatment.

## Discussion

The DoS ink materials and various electronic devices development and evaluations set the foundation for DoS electronics, a novel bioelectronics platform. The inclusion of semiconductor and dielectric inks that are drawable on skin enables the development of active electronics, directly on skin. Immunity to motion artifacts is a substantial advancement for bioelectronics and suggests the daily usability of DoS electronics, especially in low-resource areas. The capability of point-of-care treatment further emphasizes the versatility of DoS electronics. In addition, DoS electronics is relatively unaffected in the presence of sweat, more resistant to physical damage compared to the existing wearable bioelectronics, and moves with and adheres to the skin as it deforms, resulting in motion artifact-less sensing. The inks, pens, and stencils serve as a toolkit for simple construction of various customizable DoS electronics on textured skins, which are mechanically deformable and have curvilinear shape. The simplicity of the drawing process enables easy access to individuals of any background or knowledge level to create DoS electronics on demand. With further optimization of functional ink materials, sensor/device constructions and performances, studies of the sensor/device variability, and implementation of functionalities such as wireless read-out and communication capabilities, DoS electronics can be implemented as a new, simple, and easily accessible yet promising personalized bioelectronics and healthcare tool.

## Methods

### Materials

Ag flakes (10 µm size, 99.9% trace metals basis, 327077), regioregular P3HT (445703), tetrahydronaphthalene (522651), cyclohexanone (398241), Triton X-100 (X100), 1-ethyl-3-methylimidazolium bis(trifluoromethylsulfonyl)imide ([EMIM][TFSI], >98%), poly(vinylidene fluoride-co-hexafluoropropylene) (PVDF-HFP, 427160), and (3-aminopropyl)triethoxysilane (APTES, 440140) were purchased from Sigma Aldrich and used without further modification. PEDOT:PSS (PH 1000) was from Ossila Limited. Acetone was from Fisher Chemical. PDMS rubber (Sylgard 184 Silicone Elastomer Kit) was from Dow Corning.

### Conductive ink preparation

The Ag-PEDOT:PSS ink was prepared by first making the highly conductive PEDOT:PSS solution and then adding in the Ag flakes. First, the highly conductive PEDOT:PSS solution was made by stirring 1 wt% Triton X-100 into stock PEDOT:PSS solution for 12 h at room temperature (~22 °C)^50^. Afterward, the PEDOT:PSS was stored at ~4 °C in a refrigerator. Prior to adding Ag flakes, the PEDOT:PSS was taken out of the refrigerator and allowed to stir until the solution reached room temperature. Then the corresponding amount of Ag flakes (at 1: 2 weight ratio, Ag flakes: PEDOT:PSS solution) in the form of a powder was added to the vial, the PEDOT:PSS solution was dropped in, and the mixture was stirred on a magnetic stirrer for 30 min. The resulted ink was ready to use after 30 min but it could be stirred longer if there was any visible Ag powder remaining. The 1: 2 ratio showed the optimal combination of low resistance and minimal resistance change after mechanical stretch, as shown in Supplementary Fig. 7.

### Semiconducting and dielectric inks preparation

The P3HT-NF ink solution was prepared by mixing two solvents, tetrahydronaphthalene and cyclohexanone, at a 3:7 volume ratio. The mixed solvent strategy combined a good P3HT solvent (THNT) with a poor P3HT solvent (CH) to promote the formation of stable nanofibrils through increased π-π interactions in the main chains of the P3HT polymer^51^. Thereafter, 5 mg/mL of regioregular P3HT was added and the solution was stirred at 80 °C for 12 h in the dark. Afterward, the solution was placed into a −20 °C freezer for 30 min to ensure P3HT-NF formation^51,52^. The semiconducting ink was ready to use after freezing. The ion gel ink was prepared by mixing PVDF-HFP, [EMIM][TFSI], and acetone at a weight ratio of 1: 4: 7^53^. First, the PVDF-HFP was dissolved in acetone at 60 °C for 1 h and then the [EMIM][TFSI] was added. The ion gel ink was then stirred overnight at 80 °C. After that, the ion gel ink was ready to use.

### Fabrication of DoS electronics on the skin replica

The transistors were prepared using the DoS electronics drawing process. The source and drain electrodes were first drawn with the Ag-PEDOT:PSS ink onto the skin replica using the stencil (Supplementary Fig. 13, left) and left to dry for 3–5 min at room temperature. After the ink was dry and the stencil was removed, another stencil (Supplementary Fig. 13, middle) was placed on the skin replica to control the areal coverage of the drawn P3HT-NF, which dried for 5 min at room temperature. The dielectric layer of ion gel was then drawn onto the channel between the source and drain electrodes of the transistor using its corresponding stencil (Supplementary Fig. 13, right). The strain sensors, temperature sensors, and heaters were fabricated using their respective stencils (Supplementary Figs. 15 and 17) and a similar fabrication procedure. A wet paper towel and regular hand soap could be used to wipe off the DoS electronics from the skin. It should be noted that transistors (or any semiconductor devices) directly drawn on human skin may be minimally affected by the electrical potentials of the skin. In that case, an insulation layer could be placed on the skin first and then the DoS transistor could be fabricated on the insulation layer.

### Characterization of DoS stretchable electronic materials and devices

Flat PDMS substrates were made using a similar procedure described in the skin replica fabrication (see the Supplementary Information). The Ag-PEDOT:PSS and P3HT-NF inks were drawn onto the flat PDMS for mechanical characterization. SEM (XL-30S FEG, Philips) and AFM (MFP-3D ORIGIN+, Asylum Research) images were taken to visualize the morphologies and structures of the Ag-PEDOT:PSS and P3HT-NF inks, respectively. I-V characteristics were measured using a semiconductor characterization system (2612B, Keithley). Stretching/releasing tests were performed with a cyclic automatic stretcher (CK-700FET, CKSI Co. Ltd.). A power supply (1627A, BK Precision) was used to apply DC bias to the DoS heater.

### EP data acquisition

The areas of skin on which the electrodes were placed were prepared with an alcohol prep pad that was scrubbed on the skin for a few seconds. It is noted that this preparation step is not always necessary to successfully capture EP signals. DoS EP sensors were fabricated by drawing with the pen containing Ag-PEDOT:PSS directly on skin. The EP sensors were ~ 15 × 15 mm. Double-sided conductive acrylic tape (ARclad® 8001–77, Adhesives Research), cut to approximately the same dimensions as the DoS EP sensor, was laminated onto the sensors, and electrical leads were attached to the conductive tape. Gel electrodes (Meditrace 450 Foam Electrodes, Kendall) were placed next to the DoS EP sensors, and as with the previous, snap electrical leads were attached. To connect the mesh electrodes to the DAQ setup, first, a layer of single sided tape (Magic Tape, 3 M) was laminated to the skin, then a layer of conductive tape was laid on top. The mesh electrode was placed on the skin and the contact pads of the electrode were attached to the conductive tape. Then, snap electrode cables were affixed to the conductive tape. The schematic of this interface is shown in Supplementary Fig. 28. The snap electrical leads were connected to an interface board (RHD2000, Intan Technologies) via an amplifier board (RHD2216, Intan Technologies) with bipolar input channels. To measure ECG, two of each electrode type were drawn/placed on the right (- electrode wire) and left (+ electrode wire) wrists (Supplementary Fig. 21a) and were connected to the amplifier board. In the DAQ program, a sampling rate of 2000 Hz was utilized^41,42^ and the notch filter (60 Hz) setting was turned on. The ground lead was placed on the elbow of a subject. To measure EMG, two electrodes were placed on the same arm, with one on the forearm and another on the bicep (Supplementary Fig. 21b). The same program settings were used to acquire EMG, apart from the bandwidth which was set to 0.1–1000 Hz. No further post processing was done with the data acquired from this wired acquisition setup for any of the experiments.

### Wireless DoS EP monitoring system

The circuit consisted of an amplifier, microcontroller, Bluetooth module, and a 3.7 V battery (Fig. 3j) and the circuit schematic is shown in Supplementary Fig. 24a. The electrical leads from the amplifier were placed onto a conductive adhesive (ARclad® 8001–77, Adhesives Research) that was laminated onto the DoS EP sensors (Supplementary Fig. 24b). The microcontroller programmed in Arduino was used to capture the ECG data via Bluetooth and display in real-time (Supplementary Fig. 24c). It is noted that the data acquired with the wireless ECG monitoring system could easily be transmitted to any Bluetooth compatible device. As a demonstration for single-arm ECG capture, the DoS EP sensors were placed on the skin of the upper left arm, specifically near the underside of the arm for ground, the triceps for the - side, and the biceps for the + side. The electrodes were connected using the appropriate interface to a mounted circuit comprised of a microcontroller (Arduino Nano, Arduino), an amplifier board (AD8232, Sparkfun), a Bluetooth module (HC-05, DSD TECH), and a 3.7 V Li-po battery. Arduino and Processing scripts for capturing ECG in the online Sparkfun AD8232 heart rate monitor hookup tutorial were utilized. The Arduino script was modified to account for Bluetooth transmission and was uploaded to the microcontroller. After the circuit was powered on, it was connected to the computer via Bluetooth. The real-time wireless data was visualized and acquired by running the Processing script. The original script was modified to save data with time stamps into a spreadsheet. Further post processing in MATLAB involved removing outliers using an adaptive median filter through detection of R peaks, high-pass filtering (cutoff frequency of 1 Hz) with a 1st order butterworth filter to shift the signal baseline to zero and converting the analog-to-digital converter (ADC) bit values into voltage. It should be noted that the amplifier used for the wireless acquisition did not have the gain factored out of the output data, so the gain (100) was factored out during post processing.

For the simple stress test, the electrodes were drawn on the right side of the chest (− side), left side of the chest (+ side), and on the right hip (ground). The circuit was mounted onto the subject’s abdomen with a waist trimmer. The subject was instructed to stand still for 16 s, then walk for 16 s. This exercise was repeated 10 times to get the average HR data. Walking was done in rhythm to a metronome set to 120 bpm which roughly equated to walking at 5 km/h. The subject paced 2 m from and back to the computer that was acquiring the data via Bluetooth.

To obtain the HR data, the R-R intervals were calculated from the filtered ECG signals through R peak detection. Dividing 60 s by the R-R interval gave the HR in bpm.

### Sweat, durability, and adhesion tests

Two sensors of the three sensor types (DoS, gel, and mesh) were affixed as shown in Supplementary Fig. 21b for measuring ECG using the wired DAQ (RHD2000, Intan Technologies) setup. Prior to sweating, the ECG was recorded. Then, the subject walked intensely for 15 min outdoors (~38 °C) and their ECG was recorded again after they completed the exercise.

The durability tests were conducted using the same setup, except that the subject continued regular activities, and had the ECG recorded after 4 and 7 h. Again, the wired DAQ setup was utilized to acquire the ECG data. The ECG signals attained with the DoS EP sensors were relatively consistent over all the measurement time points (Supplementary Fig. 30a). The amplitude of the ECG signals obtained with the gel electrodes decreased (Supplementary Fig. 30b), which is due to the gel drying out. ECG signals attained with the mesh electrodes showed increased noise at hour 7 (Supplementary Fig. 30c). Greater noise could be attributed to partial delamination that occurred from physical rubbing of the mesh electrode during daily activities.

For the peeling tests, a piece of clear tape (Magic Tape, 3M) was placed on top of the DoS EP sensor, conductive gel from the gel electrode, and mesh electrode and then removed. The gel electrode was peeled off using a tensile tester (Mark-10 with digital force gauges Series 5). As shown in Supplementary Fig. 31a, the DoS EP sensor cannot be removed from the skin and only excess Ag flakes are barely noticeable on the removed tape. The gel electrodes strongly adhere to the skin due to the additional adhesive surrounding the conductive gel (Supplementary Fig. 31b). Long-term use of the gel electrodes resulted in difficulties in removal and created skin irritation for one subject (Supplementary Fig. 31c), as also reported elsewhere^6^. If the conductive gel was removed from the gel electrode and placed on the skin, it could easily be removed with a piece of tape (Supplementary Fig. 31d), which suggests that motion artifacts are generated by movement at the gel and skin interface due to improper adhesion^36,37^. The same observation was made with the mesh electrodes, as shown in Supplementary Fig. 31e.

The rubbing tests were performed by placing one finger on top of the sensor and rubbing back and forth intensely for a few seconds. It is noted that, as aforementioned, any imperfections in the DoS devices could easily be fixed, whereas an entirely new device would be necessary for the damaged mesh electrodes.

### Skin deformation induced motion during EP sensing

For the skin deformation experiment, two sensors from each of the three sensor types were individually affixed to the skin using the same positions used for recording ECG with the wired DAQ system. During recording, two fingers were placed ~ 1 cm on each side of one sensor as shown in Supplementary Fig. 33b. The skin was stretched/compressed for about 4–6 s. The duration of the stretching/releasing/compressing motion was determined to be 1 s based on markers in the recorded data, and the speed of the applied deformations was determined to be about 2 mm/s. Stretching and compressing were done perpendicular to the length of the arm.

### Vibrating motor-induced motion during EP sensing

Two sensors of each of the three types were affixed to the skin and were placed 20 cm apart as shown in Supplementary Fig. 41. A 10 mm diameter VM (HUELE micro-motor) was attached to the skin using regular double-sided tape. It was placed between and equidistant from each of the two sensors. The VM was connected to a function generator (DG4062, RIGOL) set to 1000 Hz, 5 V_pp_, 1.5 V DC offset, and ramp waveform. The output of the function generator was turned on and off to control motor-induced vibration. The vibration frequency of the VM was verified by applying lower frequencies (<10 Hz) that could be easily perceived and counted. TF analysis was performed in MATLAB using a window length of 50, 75% overlap, and Hamming window type for all the sensor types. The first supplemental experiment was performed by increasing the DC offset to 3 V (Supplementary Fig. 43a) and thereby increasing the vibration amplitude. The second supplemental experiment involved moving the VM closer to and on top of one of the DoS EP sensors (Supplementary Fig. 43b–e).

### Wound healing

First, three Cd-1 mice were anesthetized with isoflurane. Dorsal hair was removed with hair removal cream and a wound ~ 1 cm wide was surgically generated by cutting off the epidermis while maintaining the dermis so that the wound would not shrink too much. On days 1 and 3, electrodes were drawn on both sides of the wound (to mimic capacitor plates) and pulsed DC stimulation (30 µA DC pulses, 100 µs in duration, every 15 ms) was applied for 1 h on each of those days. The schematic experimental setup is shown in Supplementary Fig. 44. On day 5, samples of the wound were harvested. Hematoxylin and eosin (H&E) stain was utilized for the histology. Images shown were all taken from the same mouse. All procedures followed the National Institutes of Health Guide for the Care and Use of Laboratory Animals and were approved by the University of Chicago Institutional Animal Care and Use Committee (Protocol 72219).

## Supplementary information

Supplementary Information

## Data Availability

The authors declare that all data supporting the findings of these studies are available within the paper and its Supplementary Information.

## Code availability

Custom code used to analyze the data are available from the corresponding author upon reasonable request.
