# Supplementary material for: Ultra-conformal drawn-on-skin electronics for multifunctional motion artifact-free sensing and point-of-care treatment
Source: Nat Commun. 2020 Jul 30;11:3823. doi: 10.1038/s41467-020-17619-1 (PMC7393123; doi:10.1038/s41467-020-17619-1)
Supplement: Supplementary file 1 — Supplementary Information [file 41467_2020_17619_MOESM1_ESM.docx]

**Supplementary Information**

**Ultra-Conformal Drawn-on-Skin Electronics for Multifunctional Motion Artifact-Free Sensing and Point-of-Care Treatment**

Faheem Ershad^1^, Anish Thukral^2^, Jiping Yue^3^, Phillip Comeaux^1^, Yuntao Lu^4^, Hyunseok Shim^4^, Kyoseung Sim^2^, Nam-In Kim^4^, Zhoulyu Rao^4^, Ross Guevara^1^, Luis Contreras^1^, Fengjiao Pan^2^, Yongcao Zhang^4^, Ying-Shi Guan^2^, Pinyi Yang^2^, Xu Wang^4^, Peng Wang^2^, Xiaoyang Wu^3^, Cunjiang Yu^1,2,4,5,6,*^

^1^ Department of Biomedical Engineering, University of Houston, Houston, TX, 77204, USA

^2^ Department of Mechanical Engineering, University of Houston, Houston, TX, 77204, USA

^3^ Ben May Department for Cancer Research, The University of Chicago, Chicago, IL, 60637, USA

^4^ Materials Science and Engineering Program, University of Houston, Houston, TX, 77204, USA

^5^ Department of Electrical and Computer Engineering, University of Houston, Houston, TX, 77204, USA

^6^ Texas Center for Superconductivity, University of Houston, Houston, TX, 77204, USA

*Correspondence to: cyu15@uh.edu

**Supplementary Figures**

**
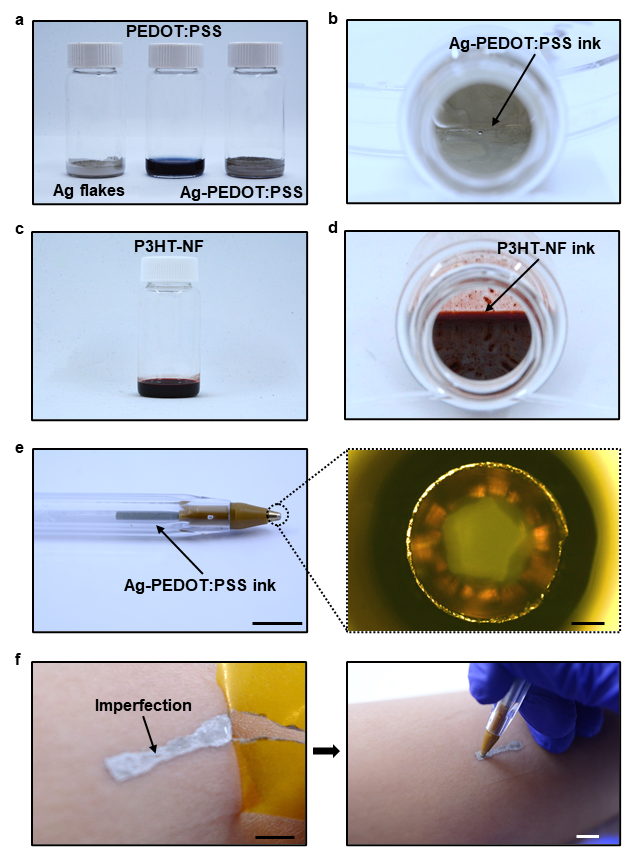
**

**Supplementary Fig. 1. Conductive and semiconducting inks used with the ballpoint pen. a,** From left to right, images of the vials containing Ag flakes, PEDOT:PSS, and Ag-PEDOT:PSS ink. **b,** Image of the thoroughly mixed Ag-PEDOT:PSS ink in a vial. **c,** Vial containing P3HT-NF ink. **d,** Dispersed P3HT-NF inside of the vial. **e,** Images of the conductive ink loaded in a pen (left, scale bar 1 cm) and optical microscope image of the tip (right, scale bar 200 µm). **f,** An imperfection (left frame, scale bar 5 mm) corrected with the Ag-PEDOT:PSS ink (right frame, scale bar 1 cm).


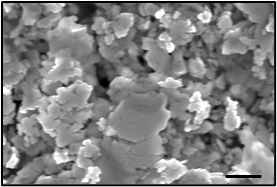


**Supplementary Fig. 2. SEM image showing the nano/micro-flake structures of the Ag-PEDOT:PSS ink (scale bar 2 µm).**

**
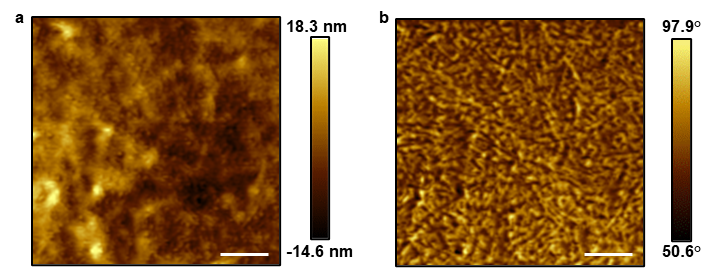
**

**Supplementary Fig. 3. AFM Images of the P3HT-NF film. a,** AFM surface topography of the P3HT-NF film (scale bar 500 nm). **b,** Phase mode image of the P3HT-NF film (scale bar 500 nm).

**
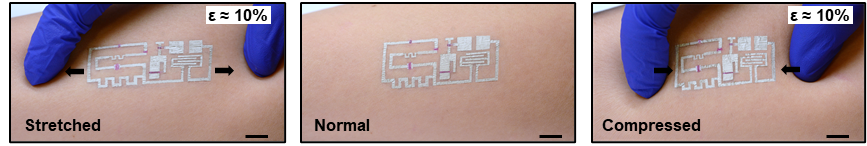
**

**Supplementary Fig. 4. Stretching and compressing the multifunctional prototype DoS circuit made from the Ag-PEDOT:PSS, P3HT-NF, and ion gel inks (scale bars 1 cm).**

**
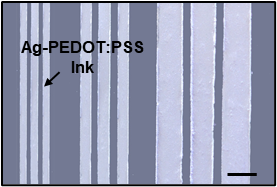
**

**Supplementary Fig. 5.** **Control over the line width of the Ag-PEDOT:PSS ink by varying the pen tip diameter.** From left to right, the line widths are 0.3 mm, 0.5 mm, and 1 mm (scale bar 1 mm).

**
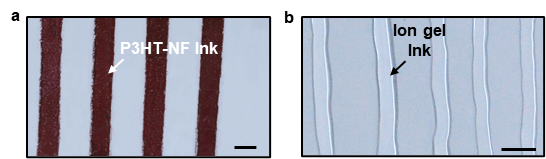
**

**Supplementary Fig. 6. Images of drawn P3HT-NF and ion gel inks.** **a,** Drawn lines of the P3HT-NF ink (scale bar 1 mm). **b,** Drawn lines of the ion gel ink (scale bar 5 mm).

**
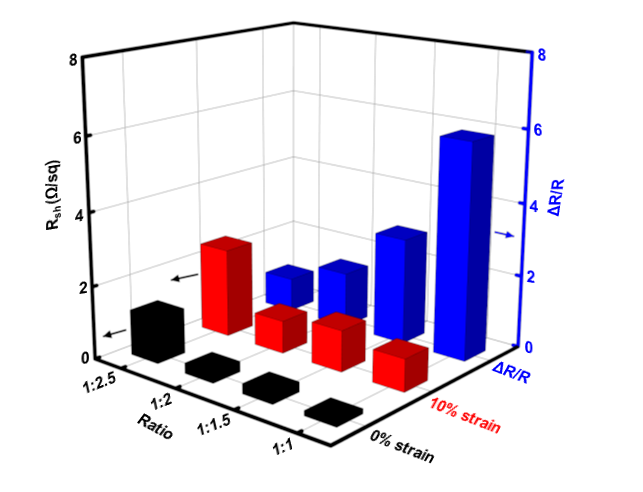
**

**Supplementary Fig. 7. Comparison between the sheet resistances, stretchability, and change in resistance of different Ag flakes : PEDOT:PSS ink ratios.**

**
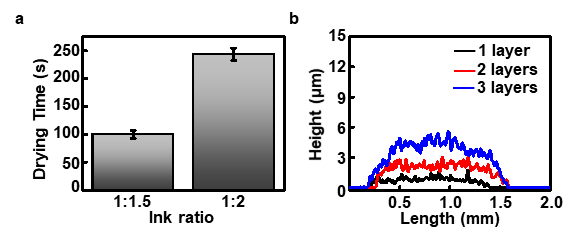
**

**Supplementary Fig. 8. Characteristics of Ag-PEDOT:PSS ink. a,** Time required for two different Ag-PEDOT:PSS ink ratios to visibly dry. **b,** Thickness obtained with a profilometer of the Ag-PEDOT:PSS ink based on drawing multiple ink layers.

**
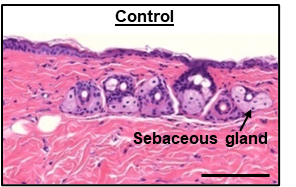
**

**Supplementary Fig. 9. Histological image of the harvested control skin sample from mice (scale bar 100 µm).**

**
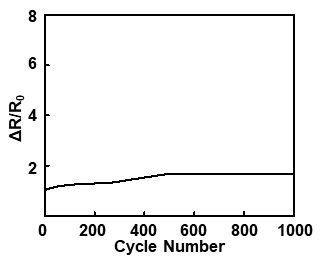
**

**Supplementary Fig. 10. Normalized resistance change of Ag-PEDOT:PSS ink after stretching for 1000 cycles at 10% strain.**

**
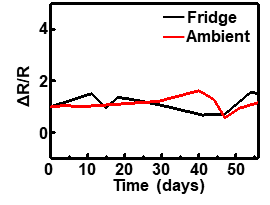
**

**Supplementary Fig. 11. Ag-PEDOT:PSS ink storage.** Effect of storing the Ag-PEDOT:PSS ink at room temperature and fridge (~ 4℃) on the ink’s electrical resistance.

**
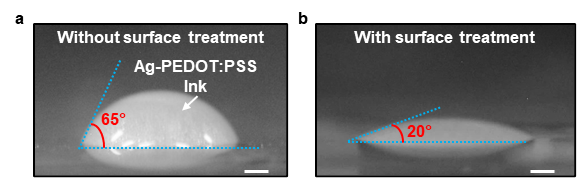
**

**Supplementary Fig. 12. Comparison of contact angle of Ag-PEDOT:PSS on PDMS depending on the surface treatment. a,** Contact angle before APTES treatment (scale bar 500 µm). **b,** Contact angle after APTES treatment (scale bar 500 µm).

**
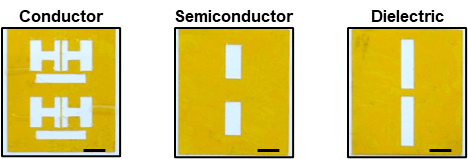
**

**Supplementary Fig. 13. Stencils for fabricating the DoS transistor.** The Ag-PEDOT:PSS ink is drawn into the transistor stencil (left). The P3HT-NF ink is drawn into the semiconductor stencil (middle). The ion gel ink is drawn into the dielectric stencil (right). The stencils are removed after the corresponding ink layer is dried (scale bars 5 mm).


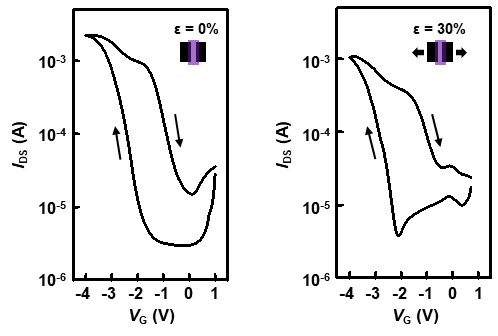


**Supplementary Fig. 14. Hysteresis of the transfer characteristics of DoS transistors without strain (left) and under 30% strain (right).**

**
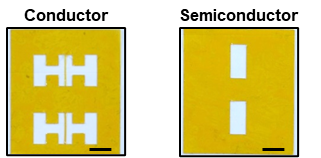
**

**Supplementary Fig. 15. Stencils for fabricating the DoS strain/temperature sensor.** The Ag-PEDOT:PSS ink is drawn into the strain/temp. stencil (left). The P3HT-NF ink is drawn into the semiconductor stencil (right). The stencils are removed after the corresponding ink layer is dried (scale bars 5 mm).

**
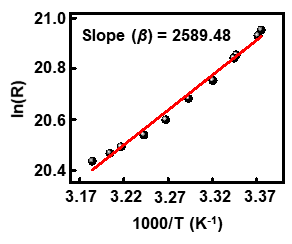
**

**Supplementary Fig. 16. Linear plot showing ln(R) versus 1000/T of the DoS temperature sensor.**

**
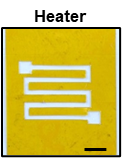
**

**Supplementary Fig. 17. Stencil for fabricating the DoS heater using the Ag-PEDOT:PSS ink (scale bar 5 mm).**

**
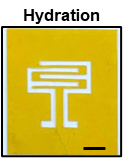
**

**Supplementary Fig. 18. Stencil for Ag-PEDOT:PSS based DoS skin hydration sensor (scale bar 5 mm).**

**
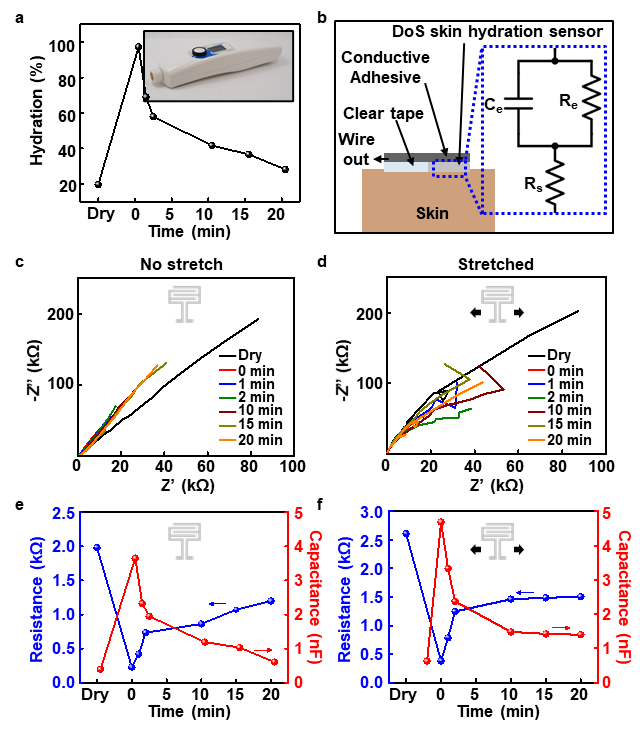
**

**Supplementary Fig. 19. Characterization of the DoS skin hydration sensor. a,** Commercial skin hydration meter measurements of dry and moisturized skin**. b**, Schematic circuit diagram showing the DoS hydration sensor and skin interface. **c,** Nyquist plots obtained with the DoS skin hydration sensor at different measurement time points with the device under no strain. **d,** Nyquist plots obtained with the DoS hydration sensor at different measurement time points with the device gently stretched on the skin. **e,** Equivalent resistance (R_e_) and capacitance (C_e_) without strain applied to the DoS skin hydration sensor. **f,** Equivalent resistance (R_e_) and capacitance (C_e_) with strain applied to the DoS skin hydration sensor.

**
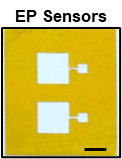
**

**Supplementary Fig. 20. Stencil for Ag-PEDOT:PSS based DoS EP sensors (scale bar 5 mm).**

**
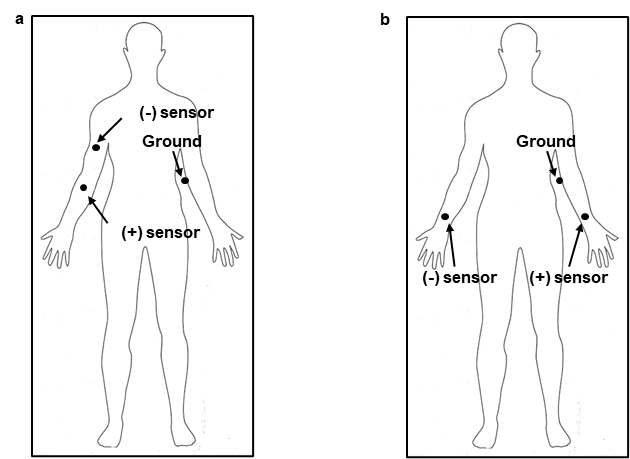
**

**Supplementary Fig. 21. EMG and ECG sensor placements. a,** Schematic of EMG sensor placement on the right forearm and upper arm. **b,** Schematic of ECG sensor placement on the wrists.


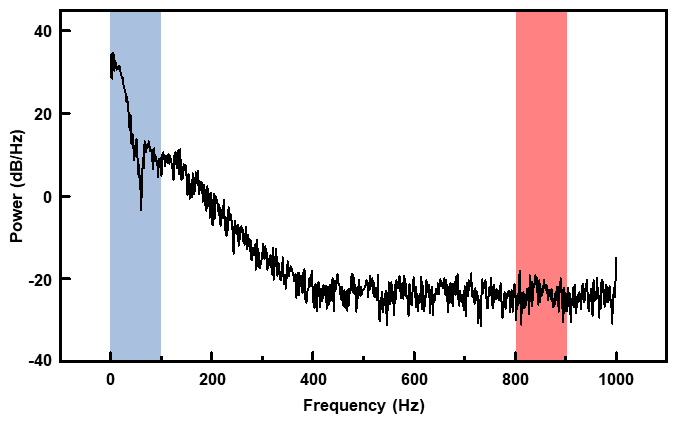


**Supplementary Fig. 22. Power spectral density of ECG signals attained using DoS EP sensors.** The blue region indicates the relevant ECG frequencies and the red region indicates the frequencies in the noise floor.


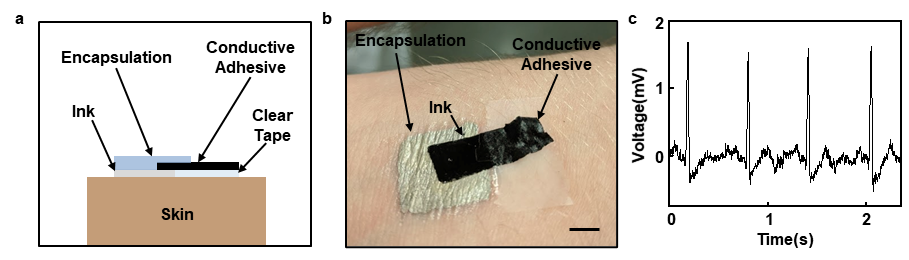


**Supplementary Fig. 23. Encapsulated DoS EP sensors. a,** Cross section schematic of the encapsulated DoS EP sensor. **b,** Camera image of the DoS EP sensor encapsulated with liquid bandage on the wrist of a human subject. Liquid bandage (New-Skin) was spread on top of the DoS EP sensor (scale bar 5 mm). **c,** ECG signals recorded using the encapsulated DoS EP sensors.


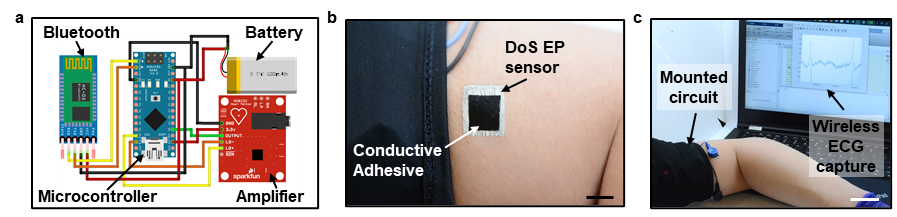


**Supplementary Fig. 24. Wireless EP monitoring setup. a,** Diagram of the circuit created using Fritzing software. **b,** Photo of the interface without the snap electrical lead (scale bar 1 cm). **c,** Image of the mounted circuit on the arm and ECG signal acquisition via Bluetooth (scale bar 5 cm).


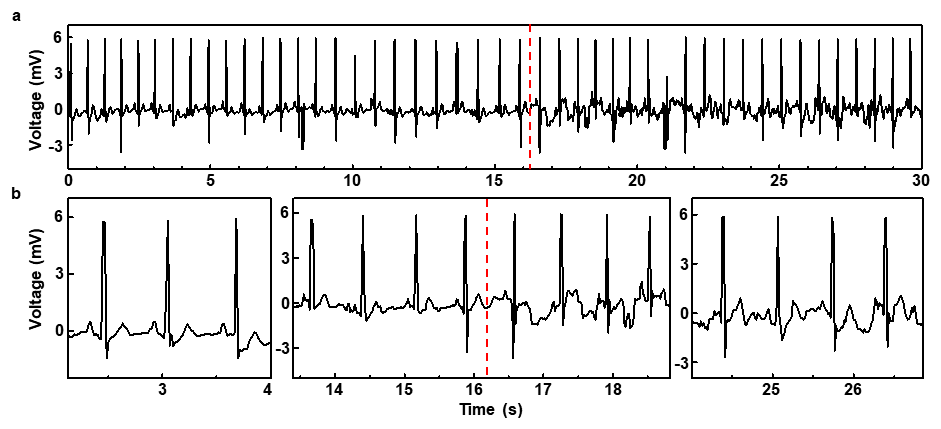


**Supplementary Fig. 25. Wireless EP monitoring stress test. a,** Representative trial of walking with the wireless transmission circuit connected to DoS EP sensors for recording ECG. Prior to the red dashed line, the subject was standing still. After the red dashed line, the subject began walking. **b,** The left frame shows the ECG signal when the subject was standing, the middle frame shows the ECG signals when the subject transitioned between standing and walking, and the right frame shows the ECG signals when the subject was walking.


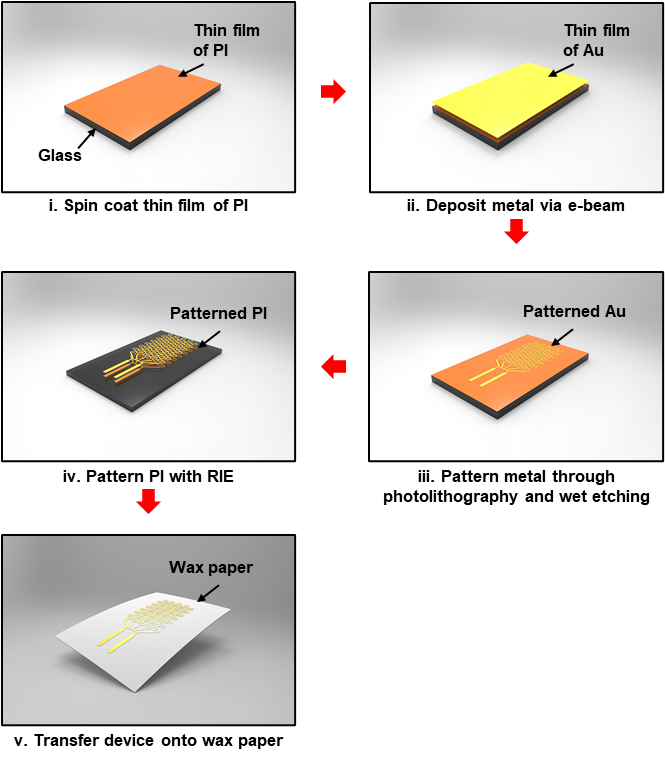


**Supplementary Fig. 26. Fabrication process for the ultrathin serpentine gold mesh electrodes.**


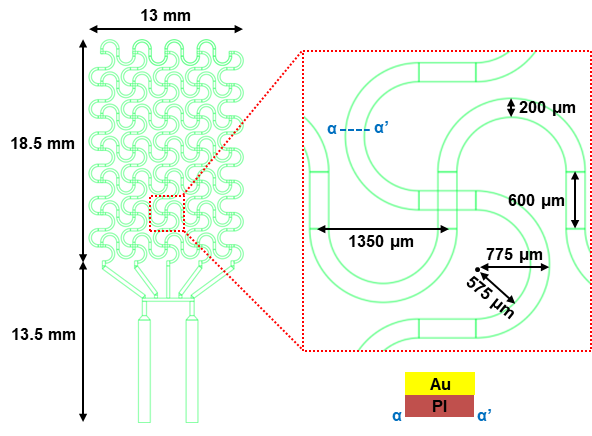


**Supplementary Fig. 27. Detailed geometrical parameters for mesh electrodes.**


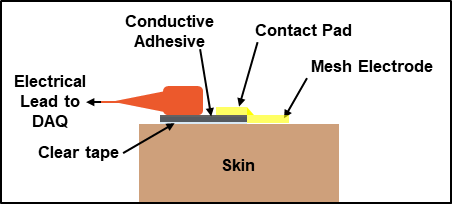


**Supplementary Fig. 28. The side view schematic of the interface between the skin and mesh electrodes.**

**
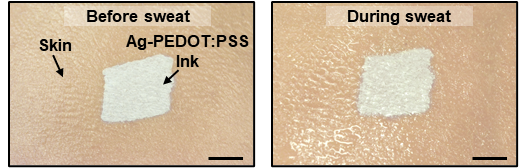
**

**Supplementary Fig. 29. Optical image of the Ag-PEDOT:PSS ink drawn on the forehead before sweat (left) and during sweat (right, scale bars 5 mm).**


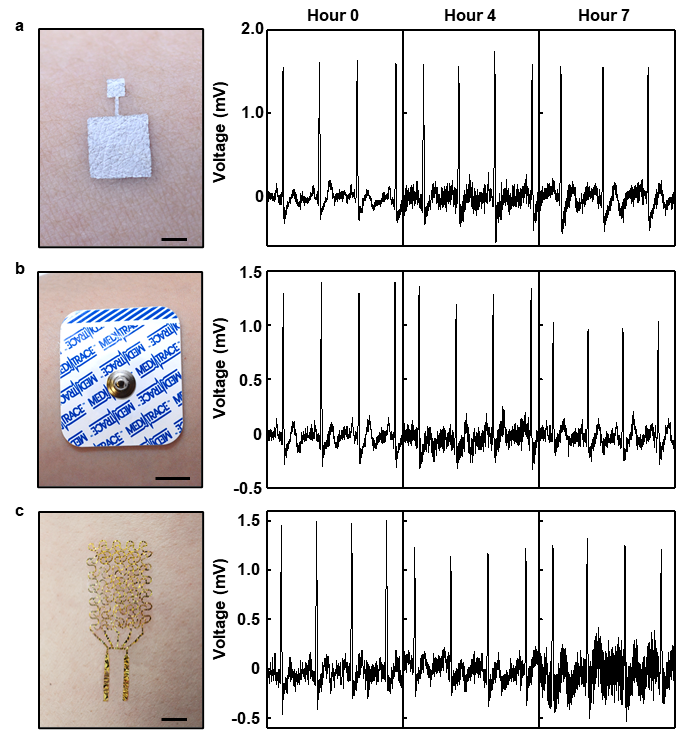


**Supplementary Fig. 30. Sensor durability over time for recording ECG. a,** ECG recordings attained with DoS EP sensors (left frame, scale bar 2 mm) over 7 hrs (right frames)**. b,** ECG recordings attained with gel electrodes (left frame, scale bar 1 cm) over 7 hrs (right frames). **c,** ECG recordings attained with mesh electrodes (left frame, scale bar 5 mm) over 7 hrs (right frames).


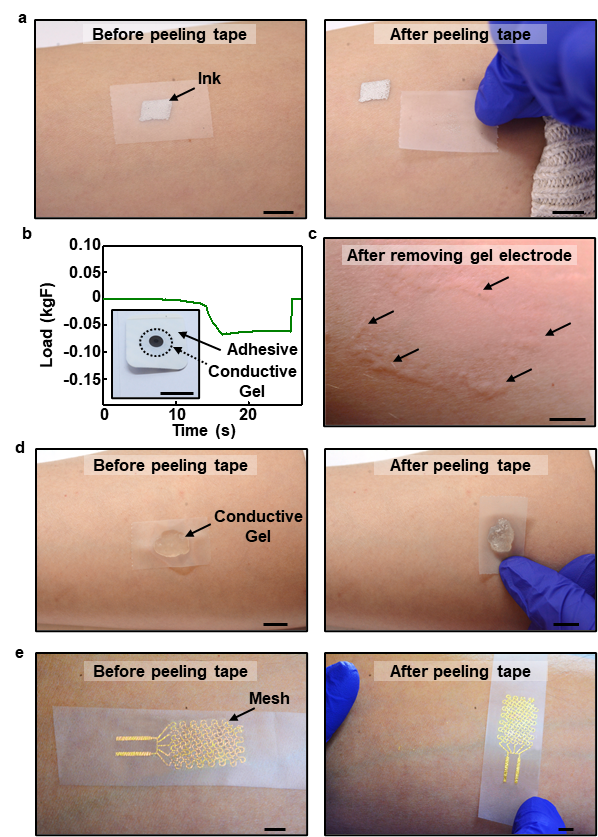


**Supplementary Fig. 31. Comparison of sensor adhesion to skin. a,** DoS EP sensor before peeling tape (left) and after peeling tape (right, scale bars 1 cm). **b,** Adhesion force test using a tensile tester for the gel electrodes (scale bar 2 cm). **c,** Skin irritation caused by the gel electrodes (scale bar 1 cm). **d,** Conductive gel removed from the gel electrode before peeling tape (left) and after peeling tape (right, scale bars 1 cm). **e,** Mesh electrode before peeling tape (left) and after peeling tape (right, scale bars 5 mm).


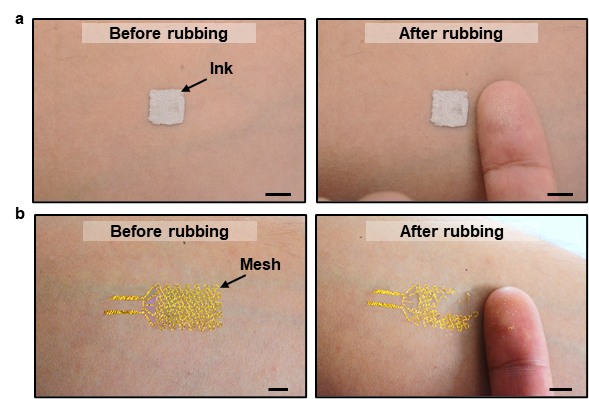


**Supplementary Fig. 32. Physical rubbing of the DoS EP sensors and mesh electrodes.** **a,** DoS EP sensor before (left) and after rubbing vigorously with one finger (right, scale bars 5 mm). **b,** Mesh electrode before (left) and after rubbing vigorously with one finger (right, scale bars 5 mm).


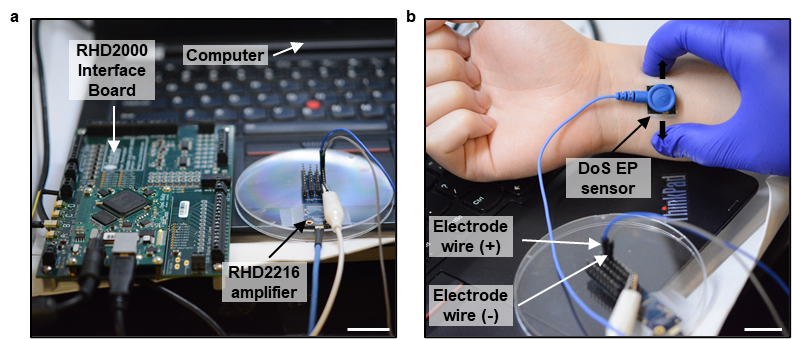


**Supplementary Fig. 33. Experimental setup for skin deformation induced motion during EP sensing. a,** DAQ setup composed of the amplifier board, interface board, and computer (scale bar 2 cm). **b,** Connection between amplifier board and the subject along with an example of applied stretching (scale bar 2 cm).


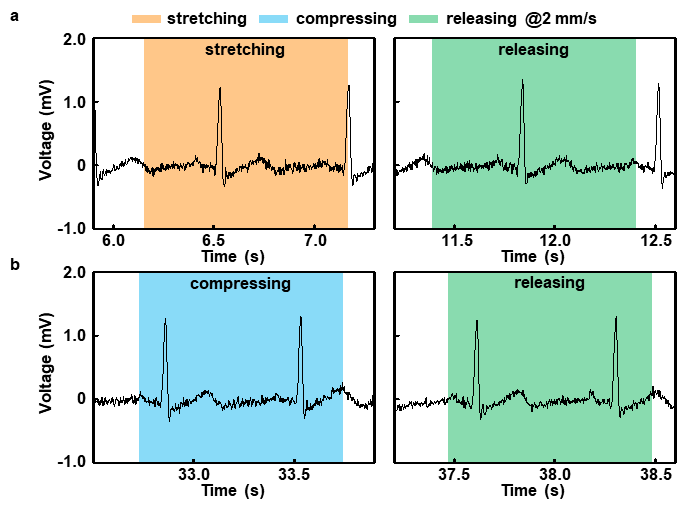


**Supplementary Fig. 34. Zoomed-in view of ECG recordings during skin deformation applied to the DoS EP sensor to generate motion artifacts. a,** Zoom in of the stretching (left) and releasing (right) cycles shown in Figure 5a. **b,** Zoom in of the compressing (left) and releasing (right) cycles shown in Figure 5a.


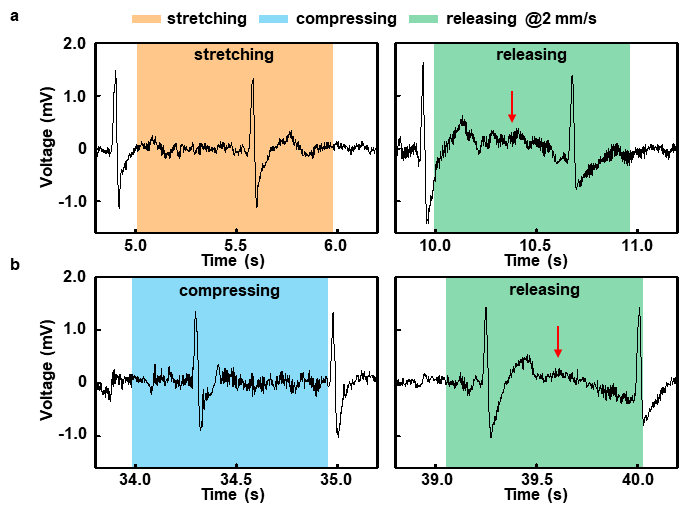


**Supplementary Fig. 35. Zoomed-in view of ECG recordings during skin deformation applied to the gel electrodes to generate motion artifacts. a,** Zoom in of the stretching (left) and releasing (right) cycles shown in Figure 5b. **b,** Zoom in of the compressing (left) and releasing (right) cycles shown in Figure 5b.


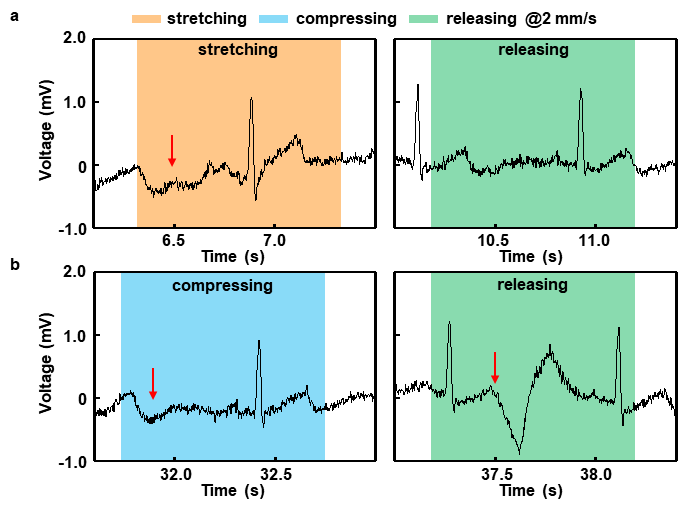


**Supplementary Fig. 36. Zoomed-in view of ECG recordings during skin deformation applied to the mesh electrodes to generate motion artifacts.** **a,** Zoom in of the stretching (left) and releasing (right) cycles shown in Figure 5c. **b,** Zoom in of the compressing (left) and releasing (right) cycles shown in Figure 5c.


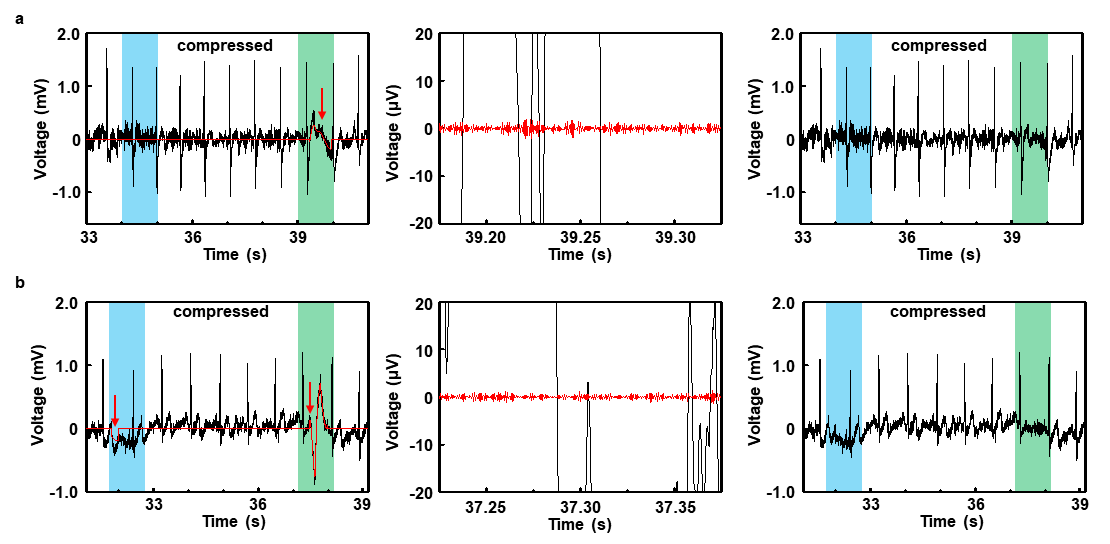


**Supplementary Fig. 37. Examples of noise and artifact removal. a,** Left frame showing the noise and artifact signal (in red) overlaying the raw ECG signal recorded with the gel electrodes. Middle frame shows a zoomed in view of the noise. Right frame shows the artifacts and noise removed from the signal. **b,** Left frame showing the noise and artifact signal (in red) overlaying the raw ECG signal recorded with the mesh electrodes. Middle frame shows a zoomed in view of the noise. Right frame shows the artifacts and noise removed from the signal.


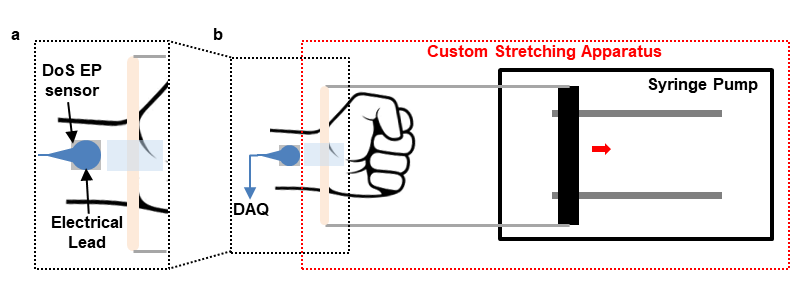


**Supplementary Fig. 38. Setup for the controlled stretch/compress deformation during EP sensing. a,** Zoomed-in view of the DoS EP sensor and electrical lead on the wrist of the subject. **b,** Setup of the custom stretching apparatus based on a syringe pump. The red arrow indicates the direction in which the custom stretching apparatus pulled the skin. The wired DAQ (RHD2000, Intan Technologies) system was used to acquire ECG data with the electrode connections on the subject’s wrists while the skin deformations were performed.

**
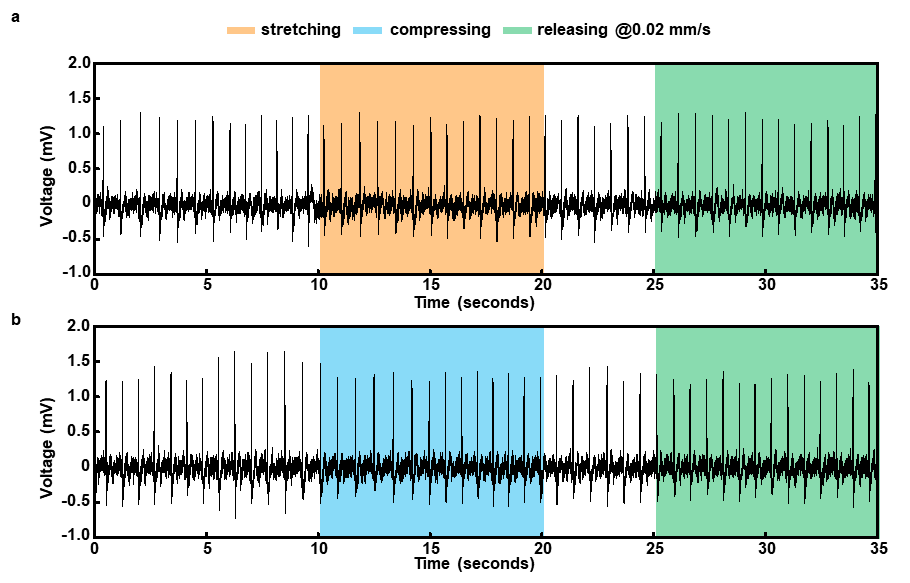
**

**Supplementary Fig. 39. ECG recordings using DoS EP sensors during controlled stretch/compress deformation. a,** ECG recorded during stretching and releasing. **b,** ECG recorded during compressing and releasing.


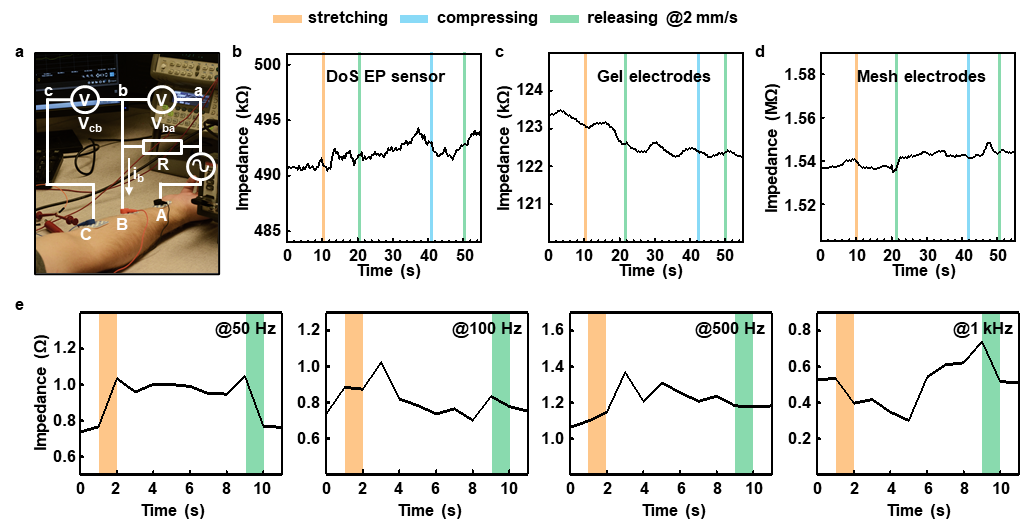


**Supplementary Fig. 40. Change in impedances during skin deformation. a,** Circuit diagram schematic of the experimental setup to acquire skin to electrode impedance (SEI). Gel electrodes were placed at positions A and C on the forearm, while B serves as the test electrode (DoS/gel/mesh). **b,** SEI of the DoS EP sensors during the stretching/releasing and compressing/releasing cycles. **c,** SEI of the gel electrodes during the stretching/releasing and compressing/releasing cycles. **d,** SEI of the mesh electrodes during the stretching/releasing and compressing/releasing cycles. **e,** Impedance recorded from the top of the DoS EP sensor on human skin at different frequencies during the stretching/releasing cycle.


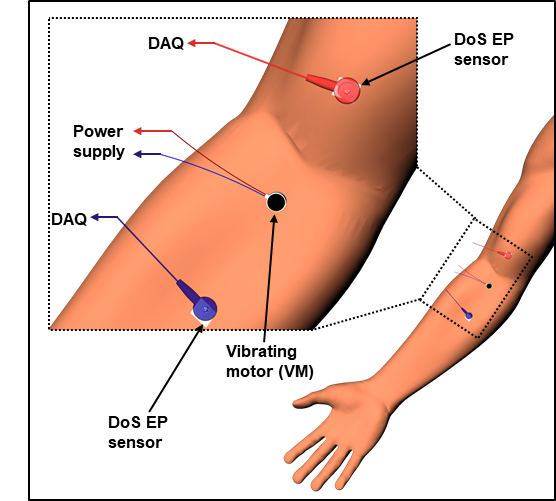


**Supplementary Fig. 41. Vibrating motor (VM) experimental setup showing the VM placed equidistant from the two sensors for recording EMG.**


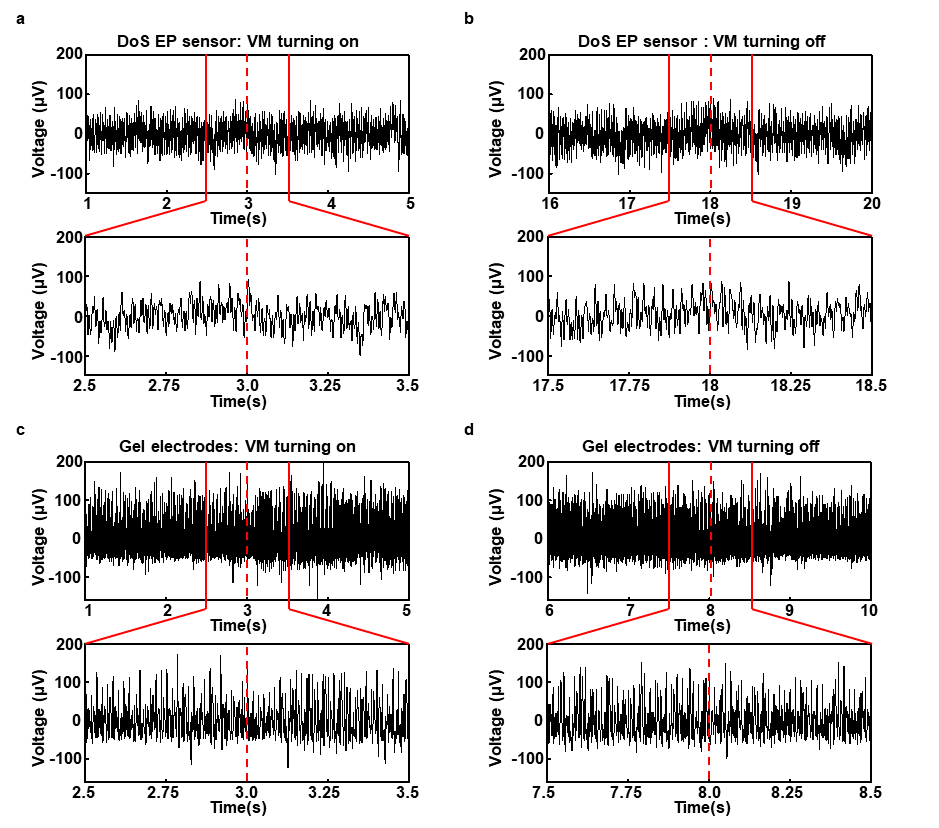


**Supplementary Fig. 42. Zoomed-in view of the VM experiment results. a,** Top frame is zoomed-in view of VM turning on as indicated by the dashed line and bottom frame is the half second time frame around the turn on point for the DoS EP sensors. **b,** Top frame is zoomed-in view of VM turning off as indicated by the dashed line and bottom frame is the half second time frame around the turn on point for the DoS EP sensors. **c,** Top frame is zoomed-in view of VM turning on as indicated by the dashed line and bottom frame is the half second time frame around the turn on point for the gel electrodes. **d,** Top frame is zoomed-in view of VM turning off as indicated by the dashed line and bottom frame is the half second time frame around the turn on point for the gel electrodes.


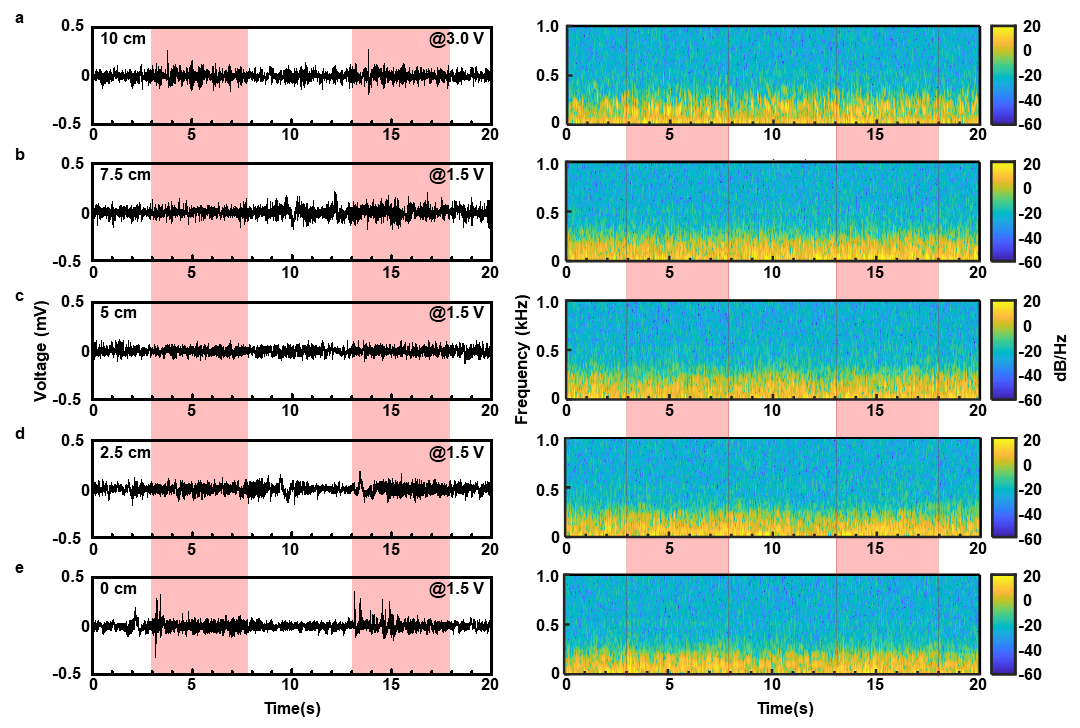


**Supplementary Fig. 43. Increased amplitude of vibration and moving the VM closer to the DoS EP sensors. a,** Resting EMG activity (left) from when the vibrating motor was set to vibrate at a higher voltage (3 V) and placed 10 cm away from the two sensors and the corresponding TF map (right). **b,** Resting EMG activity (left) from when the VM was placed 7.5 cm from one of the DoS EP sensors and the corresponding TF map (right). **c,** Resting EMG activity (left) from when the VM was placed 5 cm from one of the DoS EP sensors and the corresponding TF map (right). **d,** Resting EMG activity (left) from when the VM was placed 2.5 cm from one of the DoS EP sensors and the corresponding TF map (right). **e,** Resting EMG activity (left) from when the VM partially covered one of the DoS EP sensors and the corresponding TF map (right).


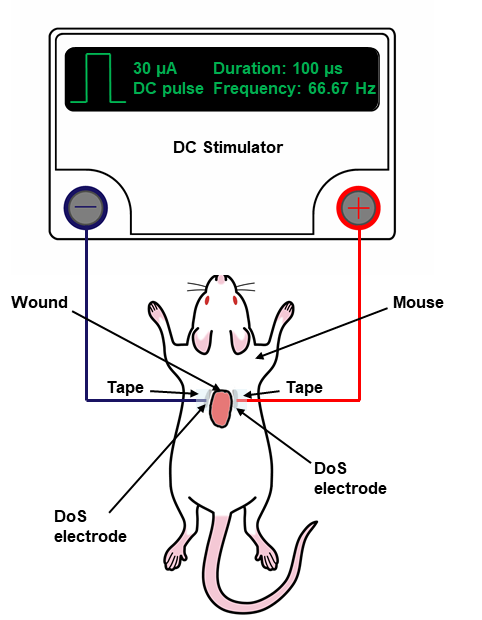


**Supplementary Fig. 44. Experimental setup for wound healing.**

**Supplementary Tables**

| **Sensor Type** | **SNR before sweating (dB)** | **SNR during sweating (dB)** | **Resistance/Contact area (Ω/mm^2^)** | **SEI/Contact area**  **(kΩ/mm^2^)** |
| --- | --- | --- | --- | --- |
| DoS | 48 | 47 | 0.263 | 2.66 |
| Gel | 48 | 45 | 1,220 | 1.95 |
| Mesh | 48 | 47 | 0.384 | 24.2 |

**Supplementary Table 1. SNR and electrical properties of the DoS EP sensor, gel electrodes, and mesh electrodes.**

**Supplementary Notes**

**Supplementary Note 1. Characteristics of the DoS thin film transistors**

The field-effect mobility ($\mu_{\mathrm{FE}}$) was calculated by fitting the plot of the linear regime of the square root of the drain current ($\sqrt{I_{\mathrm{DS}}}$) versus gate voltage (*V*_GS_) curve based on the following equations:

$I_{\mathrm{DS}}=\frac{WC_{i}\mu_{\mathrm{FE}}}{2L}(V_{\mathrm{GS}}-V_{\mathrm{TH}})^{2}$ (S1)

$\sqrt{I_{\mathrm{DS}}}=\sqrt{\frac{WC_{i}\mu_{\mathrm{FE}}}{2L}}(V_{\mathrm{GS}}-V_{\mathrm{TH}})=\sqrt{\frac{WC_{i}\mu_{\mathrm{FE}}}{2L}}V_{\mathrm{GS}}-\sqrt{\frac{WC_{i}\mu_{\mathrm{FE}}}{2L}}V_{\mathrm{TH}}$ (S2)

$Slope=\sqrt{\frac{WC_{i}\mu_{\mathrm{FE}}}{2L}}$ (S3)

$\mu_{\mathrm{FE}}=\frac{2L}{WC_{i}}(Slope)^{2}$ (S4)

where *L* and *W* are the channel length and width of the device, respectively, which are defined by the dimensions of the source and drain electrodes and the drawn P3HT channel. The *C_i_* is the capacitance per unit area of the ion gel dielectric and was found to be ~ 10 µF/cm^2^, as reported elsewhere^1^. The threshold voltage (*V*_TH_) is defined as the x-intercept from the extrapolated fitted curve.

**Supplementary Note 2. Gauge factor calculation of the DoS strain sensor.**

The change in the resistance in the P3HT semiconductor layer is defined as ΔR under applied tensile strain (*ε*). The gauge factor (*GF*) is defined by the following equation:

$GF=\frac{\Delta R/R_{0}}{\varepsilon}$ (S5)

where, *R_0_* is the resistance of the P3HT under no strain (*ε* = 0). The calculated GFs of DoS strain sensor under 10%, 20%, and 30% were 15.5, 14.6, and 15.5, respectively.

**Supplementary Note 3. DoS temperature sensor sensitivity calculations**

The change in resistance of the DoS temperature sensor can be described using the *β* parameter equation^2^:

$R=R_{\infty}e^{\frac{\beta}{T}}$ (S6)

where *R_∞_*, *β*, and *T* are the resistance at infinite temperature, thermistor constant, and absolute temperature, respectively. In the linear form of the above equation, ln*R* is directly proportional to the reciprocal of the absolute temperature (1/*T*). The plot in Supplementary Fig. 16 shows a slope of 2589.48 ± 101.75 (*β*), a y-intercept of 12.18 ± 0.33, and Pearson’s R^2^ of 0.992.

The temperature coefficient, *α*, denotes the sensitivity of NTC thermistors and is defined in the following equation^2^:

$\alpha=\frac{1}{R}\frac{dR}{dT}\times100=-\frac{\beta}{T^{2}}\times100$ (%/℃) (S7)

The calculated *α* for the temperature range (23 to 43.5℃) was -2.96 to -2.59 %/℃.

**Supplementary Note 4.** **DoS skin hydration sensor measurements**

The DoS skin hydration sensor measurements positively correlate with those of the commercial hydration meter plotted in Supplementary Figure 19a. The inset of Supplementary Fig. 19b shows the circuit diagram of the skin and device interface with R_e_ as the equivalent resistance of the skin-sensor interface in parallel with the capacitance of the interface (C_e_), both of which are connected in series with the resistance of the skin (R_s_). The interdigitated design creates finger-like capacitors between the two electrodes. The theoretical capacitance of the sensor can be controlled using the following proportional relationship:

$C_{theor} \propto Nl$ (S8)

where *C_theor_* is the theoretical capacitance of the sensor, *N* is the number of fingers in the electrode, and *l* is the length of the fingers. Increasing the number of fingers in an electrode and/or the length of these fingers results in an increased theoretical capacitance of the device^3^. The interdigitation is necessary to spread the sensing area over a wide surface area of the skin^4^. Close proximity of the interdigitated electrodes (~ 1.4 mm) results in a wider scatter field over the outer layer of the stratum corneum^5^. The distance between the electrodes was kept minimal in order to maximize sensitivity, as sensitivity has an inversely proportional relationship with the distance between the electrodes^6^.

Figure 3e shows the linear relationship (R^2^ = 0.997) described by the following equation

$\%H=bC+a$ (S9)

where *%H* is the percent skin hydration, *C* is the measured capacitance, *b* is the slope, and *a* is the calibration constant. The slope *b* and calibration constant *a* were determined to be 23.5 and 12.6, respectively. The impedance spectrums over a range of frequencies (1 kHz to 1 MHz) at different hydration conditions were recorded since they correspond to hydration levels in both the epidermis and dermis^6^. The plot in Figure 3f was generated by recording the impedance spectrums (impedance vs. frequency) for each moisture condition and then from each impedance spectrum, the impedance recorded at the 100 kHz frequency was plotted. In addition, Nyquist plots were obtained and are similar for both the non-stretched and stretched cases (Supplementary Figure 19c,d, respectively) over all measurement time points. Stretching the sensor on the skin resulted in little degradation in performance as further evidenced by the resistance and capacitance derivations (Supplementary Figure 19e,f).

**Supplementary Note 5. SNR calculation for ECG signals**

To calculate the SNR for each of the sensor types, first the power spectral density estimate was obtained using Welch’s method in MATLAB. The parameters for the pwelch function were chosen to be a 2000-point Hanning window (i.e. our sampling frequency) and a 50% overlap. The frequency range of interest for ECG signals is typically below 100-120 Hz^7,8^. As shown in Supplementary Fig. 22, the region below 100 Hz (in blue) was chosen to represent our signal in the SNR calculation and the power was summed over those frequencies and normalized to be in units of dB. The noise was averaged from the region shown in red (800-900 Hz) and this range was chosen because it was obviously in the noise floor. The following formula was used to convert the ratio of the signal and noise to power in dB:

$SNR= 10*\log_{10} \frac{P_{(s)}}{P_{(n)}}$ (S10)

where P_(s)_ is the power of the signal and P_(n)_ is the power of the noise. This approach for calculating the SNR was used for the signals shown in Figure 3i and Figure 4a-c (middle and right frames). It should be noted that if the noise was averaged across all frequencies greater than our signal of interest (i.e. >100 Hz) instead of the 800-900 Hz range, the SNR values changed negligibly.

**Supplementary Note 6. SNR calculation for ECG signals under motion**

The SNRs of the signals shown in the middle frames of Figure 5a-c were calculated using a different approach since these signals had obvious artifacts that needed to be isolated and considered as noise in the SNR calculation. The general approach to isolating the artifact was as follows. For example, the signal shown in the right frame of Figure 5b was filtered to remove most of the ECG signal and keep the artifact without attenuating too much. It should be noted that all filters utilized with butterworth filters. The raw signal was filtered with a 1^st^ order lowpass filter with a 3 Hz cutoff frequency; this will be referred to as the motion artifact signal. Simultaneously, the raw signal was filtered to isolate the noise level with a 1^st^ order bandpass filter with cutoff frequencies of 800 and 900 Hz; this will be referred to as the noise signal. These values were chosen to match the noise range utilized in the previous SNR calculations. For only the time duration for which the motion artifact occurred, the artifact was taken from the motion artifact signal and inserted into the noise signal by addition of the corresponding samples in time. This combined noise and motion artifact signal (now referred to as just the noise signal) is shown red in the left frames of Supplementary Fig. 38a,b for the gel and mesh electrodes, respectively. The noise level can be seen in the middle frames of Supplementary Fig. 38a,b. For the signals in the right frame of Figure 5c, two 1^st^ order lowpass filters were utilized (1 Hz and 20 Hz) independently to fully isolate the motion artifacts from the raw signal and the same procedure was used as just described for the gel electrodes. To demonstrate that the motion artifact isolation procedure was successful, the noise signal was subtracted from the raw signal and plotted in the right frames of Supplementary Fig. 38a,b for the gel and mesh electrodes, respectively. To calculate the SNR, the root-mean-square (rms) values of the signal (with motion artifacts and noise removed) and the rms values of the noise were divided and the following formula was utilized:

$SNR= 20*\log_{10} \frac{V_{rms(s)}}{V_{rms(n)}}$ (S11)

where V_rms (s)_ and V_rms (n)_ are the rms values of the signal (with motion artifacts and noise removed) and noise, respectively.

**Supplementary Note 7. Calculation of impedance change during local skin deformation**

To obtain the change in SEI of the test electrode at position B, gel electrodes were placed at positions A and C. The DoS EP sensor, gel electrodes, or mesh electrode served as the test electrode on the forearm. An AC signal^9^ was applied to the gel electrode at position A along with a resistor, which was connected to the test electrode at position B. The skin potentials, V_ba_ and V_cb_, were measured during stretching, compressing, and releasing motions. Two gel electrodes (at positions A and C) were utilized in order to deliver a known current and voltage through the electrode at position B. In reference to Supplementary Fig. 41a, the current between electrodes A and B can be calculated by taking the voltage difference across the known resistance of the resistor (R = 1 MΩ) as the following:

$i_{B}= \frac{V_{ba}}{R}$ (S12)

where *V_ba_* is the voltage measured by the multimeter positioned between electrodes at positions A and B, *i_B_* is the current flowing into the skin through electrode B, and *R* is the resistor placed in series with the function generator^10,11^. The input impedances of the multimeters are too high to allow flow between the electrodes through the devices, so any voltage drop between electrodes at positions B and C is taken to be due to the SEI at electrode B, *Z_B_*. This impedance is calculated based on the following equation:

$Z_{B}= \frac{V_{cb}}{i_{B}}$ (S13)

where *V_cb_* is the voltage measured by the multimeter positioned between electrodes B and C. After obtaining the SEI over time, the points at which the local deformations were applied were examined. The differences between the impedances at the start and end of the applied deformations were calculated and averaged.

The average SEI of the DoS EP sensors (492.0 ± 41.5 kΩ) was much more similar to that of the gel electrodes (122.7 ± 13.2 kΩ), unlike that of the mesh electrodes (1541 ± 3.14 kΩ) as can be seen in Supplementary Figure 40b-d, respectively. As expected, the gel electrodes generally showed the lowest average SEI due to the gel electrolyte enhancing ion conductivity, as established in prior studies^12-14^. Although it is important to consider the average SEI for electrophysiological measurements, the change in SEI during the skin deformations was of interest as it has been previously investigated as a contributor to motion artifacts in recorded signals. The change in SEI for all deformations for the DoS EP sensors, gel electrodes, and mesh electrodes were determined to be 138 ± 41.5 Ω, 54.1 ± 13.2 Ω, and 1.81 ± 1.44 kΩ, respectively. One-way analysis of variance (ANOVA) indicated that the average change in SEI for the mesh electrodes was significant (*P* = 0.03), as compared to the DoS EP sensors and gel electrodes. Post hoc analysis showed that there was no significant difference (*P* = 0.99) between the average change in SEI for the DoS EP sensors and gel electrodes. The one-way ANOVA and multiple comparisons test were performed using functions from the Statistics Toolbox in MATLAB. A threshold of *P* < 0.5 was used to determine statistical significance.

To further prove the ability of DoS sensors to accurately capture EP signals under induced motion, impedance changes on the surface of the sensor during stretching and releasing on human skin were obtained (Supplementary Figure 40e). The change in impedance at the top of the DoS EP sensor was calculated similarly. The DoS EP sensor shows very low impedance (~ 1 Ω) on top of human skin. The differences between the impedances at the start and end of the applied stretching/releasing were averaged and calculated to be 0.162 Ω.

**Supplementary Methods**

# Ballpoint Pen Preparation.

Ballpoint pens (557154012, PEN + GEAR) were fully disassembled. The balls from the pen tips and the original inks were removed. The tips and ink barrels were thoroughly cleaned in acetone, sonicated in deionized (DI) water, and air dried. Then, the Ag-PEDOT:PSS, P3HT-NF, and ion gel inks were injected into the emptied ink barrels via a syringe and 26-gauge needle.

# Stencil Fabrication.

The stencils were designed in AutoCAD. A cutting board was layered with two materials, with the top layer as Kapton tape and the bottom layer as clear single-sided tape (Magic Tape, 3M). The cutting machine (Silhouette Cameo) was programmed to cut the stencils based on the designs. The stencils were removed from the cutting board and then placed onto a sticker sheet for later use.

# DoS Prototype Multifunctional Integrated System Fabrication.

The stencil for the Ag-PEDOT:PSS ink was placed on the subject’s skin. Then the Ag-PEDOT:PSS ink was drawn into the stencil. After the ink dried, the stencil was removed. Next, small stencils were placed over the areas requiring the P3HT-NF ink, such as those for the transistor, strain sensor, and temperature sensor. For both the semiconducting and dielectric inks, it should be noted that only extremely small amounts (<0.15~0.30 µL per cm^2^) of the inks are used to contact the skin. Specifically, for a single transistor, only 0.01-0.02 µL of the solvents in the semiconducting ink and 0.1-0.2 µL of [EMIM][TFSI] in the dielectric ink were required. Once the P3HT ink dried, the stencil was removed. Finally, the ion gel ink was drawn onto the channel for the transistors. To remove the DoS integrated system from the skin, the subject used a wet paper towel with soap to rub it off. It should be noted that although the prototype is not functional, it is shown to demonstrate the wide variety of devices that can be drawn directly on the skin.

# Skin Replica Fabrication and Contact Angle Validation.

The PDMS solution was prepared by mixing the prepolymer/curing agent at the weight ratio of 10:1. Porcine skin was purchased from a grocery store and cut into pieces. On the dermis of the skin, epoxy adhesive was applied, and attached to a piece of Kapton tape. The epoxy secured the porcine skin to the tape and ensured that skin did not shrivel too much at ambient conditions. Uncured PDMS mixture was poured into a petri dish. The porcine skin pieces attached to the Kapton tape were flipped over such that the epidermis was facing down and gently submerged into the uncured PDMS. This ensured that the PDMS was thin enough for mechanical characterization. They were left to cure at room temperature for two days. Afterwards, the petri dish was put into a curing oven (~ 80℃) for one hour. After curing, the PDMS was cut, and the molds were separated from the porcine skin. Surface treatment of the skin replica was performed using UV/O_3_ for 30 min and then submerging in APTES for 10 min.

For the contact angle measurement, the sessile drop method was used based on flat PDMS substrates. The surface of the PDMS was treated using UV/O_3_ for 30 min and then submerged in APTES for 10 min.

# DoS Skin Hydration Sensor Fabrication and Characterization.

The skin hydration sensor was drawn using a stencil (Supplementary Fig. 18) onto the dry skin of the subject. The skin impedance was measured using an impedance analyzer (Multi/Autolab M204, Metrohm) connected to the DoS sensor and with commercial hydration meter (MoistureMeterSC, Delfin) prior to lotion application to serve as a comparison. Double-sided conductive acrylic tape was adhered to the contact pads of the DoS sensor as shown in Supplementary Fig. 19b. The wires from the impedance analyzer were attached to the acrylic tape. Because the adhesive was conductive, it was insulated from other parts of the skin by clear single-sided tape (Magic Tape, 3M). After the dry skin impedance measurement, the DoS sensor was then removed using a wet paper towel and regular soap. Lotion was applied to the skin for 10 s, the sensor was redrawn, and the impedance was recorded at multiple points between the time of lotion application and 20 min with the interfaced electrical leads from the impedance analyzer and commercial meter. Further details of data acquisition and plot shown in Figure 3f are described later in the Supplementary Notes.

# Fabrication of Mesh Electrodes.

First, a glass slide was cleaned using acetone, isopropyl alcohol (IPA), and DI water. A 200-250 nm thick polyimide (PI-2545, HD Microsystems) film was made by spin coating. Then 5 nm/100 nm thick Cr/Au layers were deposited via an e-beam evaporator. The metal layers were then patterned by photolithography and wet etching. The PI was patterned by reactive ion etching (RIE, Oxford Plasma Lab 80 Plus). Finally, a layer of poly(methyl methacrylate) (PMMA) was spin coated onto the metal side to aid transfer and temporarily maintain the structure of the electrode. The electrode was released from the glass using buffered oxide etchant (BOE, 6:1, Transene Company Inc.) and then picked up using a wax paper. The PMMA was dissolved using acetone. The electrode was then transferred from the wax paper to the skin.

# Controlled Stretch/Compress Deformation during EP Sensing.

A custom-made stretching apparatus based on a programmable syringe pump (BS-300, Braintree) was attached to the subject’s arm as shown Supplementary Fig. 39. Depending on the positioning of the stretching apparatus, the skin could be stretched or compressed. The syringe pump was programmed to produce a skin deformation (stretch/compress/release) at a speed of 0.02 mm/s.

# Change in SEI during Skin Deformations.

To characterize the SEI, the experimental setup shown in Supplementary Fig. 41a was utilized, as reported elsewhere^10,11^. Gel electrodes were placed at positions A and C. The sensor to be tested (DoS, gel, or mesh) was placed at position B, which was placed in between A and C, equidistant from both gel electrodes. The potential V_ba_ between electrodes A and B, and the potential V_cb_ between electrodes B and C were measured. An AC signal with a peak-to-peak potential of 6 V_pp_ (13 Hz) sine wave was delivered to the skin via electrode A^9^. A 1 MΩ resistor was connected in series with the function generator between electrodes A and B. The skin at B was manually stretched and compressed to deform the skin and the potentials were recorded over time. The measured skin potentials were converted into SEI using the equations shown in the Supplementary Notes. The average change in SEI during all deformations was then calculated for each electrode.

# Impedance Measurements from the Surface of the DoS EP Sensor.

To verify the impedance on the surface of the DoS EP sensor during stretching/releasing, two probes from an LCR meter (U1252B, Keysight) were placed 15 mm apart on a 15 x 15 mm EP sensor drawn onto the subject’s skin. AC signals with an amplitude of 1 V and frequencies ranging between 50 to 1000 Hz were applied with the LCR meter. Local deformation of the skin was introduced and the average impedance change during all deformations was calculated.

**Supplementary References**

1 Fujimoto, T. & Awaga, K. Electric-double-layer field-effect transistors with ionic liquids. *PCCP* **15**, 8983-9006 (2013).

2 Luz, F. C. S. *et al.* Construction and testing of a system for the electrical characterization of ceramic thermistors at low temperatures. *Cerâmica* **60**, 96-101 (2014).

3 Smetana, W. & Wiedermann, W. Using integrated capacitive humidity sensors in thick-film technology. *Sensors and Actuators* **11**, 329-337 (1987).

4 Fluhr, J. W., Elsner, P., Berardesca, E. & Maibach, H. I. *Bioengineering of the skin: Water and the stratum corneum*. (CRC press, 2004).

5 Sekiguchi, N. *et al.* Microsensor for the measurement of water content in the human skin. *Sensors Actuators B: Chem.* **78**, 326-330 (2001).

6 Yao, S. *et al.* A wearable hydration sensor with conformal nanowire electrodes. *Adv. Healthc. Mater.* **6**, 1601159 (2017).

7 Stauffer, F. *et al.* Skin conformal polymer electrodes for clinical ecg and eeg recordings. *Adv. Healthc. Mater.* **7**, 1700994 (2018).

8 Wang, K. *et al.* Stretchable dry electrodes with concentric ring geometry for enhancing spatial resolution in electrophysiology. *Adv. Healthc. Mater.* **6**, 1700552 (2017).

9 Talhouet, H. d. & Webster, J. G. The origin of skin-stretch-caused motion artifacts under electrodes. *Physiol. Meas.* **17**, 81-93 (1996).

10 Bergey, G. E., Squires, R. D. & Sipple, W. C. Electrocardiogram recording with pasteless electrodes. *IEEE Trans. Biomed. Eng.* **BME-18**, 206-211 (1971).

11 Spach, M. S., Barr, R. C., Havstad, J. W. & Long, E. C. Skin-electrode impedance and its effect on recording cardiac potentials. *Circulation* **34**, 649-656 (1966).

12 Cömert, A. *The assessment and reduction of motion artifact in dry contact biopotential electrodes*. (Tampere University of Technology, Tampere, 2015).

13 Hokajärvi, I. A. *Electrode contact impedance and biopotential signal quality*. (Tampere University of Technology, Tampere, 2012).

14 Lee, S. M. *et al.* Self-adhesive epidermal carbon nanotube electronics for tether-free long-term continuous recording of biosignals. *Sci. Rep.* **4**, 6074 (2014).
